# Computer-Aided Identification and Design of Ligands for Multi-Targeting Inhibition of a Molecular Acute Myeloid Leukemia Network

**DOI:** 10.3390/cancers16213607

**Published:** 2024-10-25

**Authors:** Seyedeh Sadaf Asfa, Reza Arshinchi Bonab, Onur Önder, Merve Uça Apaydın, Hatice Döşeme, Can Küçük, Alexandros G. Georgakilas, Bernhard M. Stadler, Stella Logotheti, Seyit Kale, Athanasia Pavlopoulou

**Affiliations:** 1 Izmir Biomedicine and Genome Center, 35340 Balçova, İzmir, Türkiye; sadaf.asfa@gmail.com (S.S.A.); rezaarshinchi1994@gmail.com (R.A.B.); onderonur1205@gmail.com (O.Ö.); merve.uca@ibg.edu.tr (M.U.A.); hatice.doseme@ibg.edu.tr (H.D.); seyit.kale@ibg.edu.tr (S.K.); 2 Izmir International Biomedicine and Genome Institute, Dokuz Eylül University, 35340 Balçova, İzmir, Türkiye; 3 Department of Pharmacology and Therapeutics, Max Rady College of Medicine, Rady Faculty of Health Sciences, University of Manitoba, Winnipeg, MB R3E 3P4, Canada; 4 Division of Neurodegenerative Disorders, St. Boniface Hospital Albrechtsen Research Centre, Winnipeg, MB R3E 0W2, Canada; 5 Department of Medical Biology, Faculty of Medicine, Dokuz Eylül University, 35330 Balçova, İzmir, Türkiye; can.kucuk@deu.edu.tr; 6 Physics Department, School of Applied Mathematical and Physical Sciences, National Technical University of Athens (NTUA), Zografou Campous, 15780 Athens, Greece; alexg@mail.ntua.gr; 7 Technische Hochschule Nürnberg, Faculty of Applied Chemistry, 90489 Nuremberg, Germany; Bernhard.stadler@th-nuernberg.de; 8 Biomedical Physics in Radiation Oncology, German Cancer Research Center (DKFZ), 69120 Heidelberg, Germany; styliani.logotheti@dkfz-heidelberg.de; 9 Department of Biophysics, Faculty of Medicine, Izmir Katip Çelebi University, 35330 Çiğli, İzmir, Türkiye

**Keywords:** acute myeloid leukemia, network rewiring, multi-targeting drug design, drug repurposing, DNMT3A, cyclins

## Abstract

Background/Objectives: Acute myeloid leukemia (AML) is characterized by therapeutic failure and long-term risk for disease relapses. As several therapeutic targets participate in networks, they can rewire to eventually evade single-target drugs. Hence, multi-targeting approaches are considered on the expectation that interference with many different components could synergistically hinder activation of alternative pathways and demolish the network one-off, leading to complete disease remission. Methods: Herein, we established a network-based, computer-aided approach for the rational design of drug combinations and de novo agents that interact with many AML network components simultaneously. Results: A reconstructed AML network guided the selection of suitable protein hubs and corresponding multi-targeting strategies. For proteins responsive to existing drugs, a greedy algorithm identified the minimum amount of compounds targeting the maximum number of hubs. We predicted permissible combinations of amiodarone, artenimol, fostamatinib, ponatinib, procaine, and vismodegib that interfere with 3–8 hubs, and we elucidated the pharmacological mode of action of procaine on DNMT3A. For proteins that do not respond to any approved drugs, namely cyclins A1, D2, and E1, we used structure-based de novo drug design to generate a novel triple-targeting compound of the chemical formula C15H15NO5, with favorable pharmacological and drug-like properties. Conclusions: Overall, by integrating network and structural pharmacology with molecular modeling, we determined two complementary strategies with the potential to annihilate the AML network, one in the form of repurposable drug combinations and the other as a de novo synthesized triple-targeting agent. These target–drug interactions could be prioritized for preclinical and clinical testing toward precision medicine for AML.

## Simple Summary

In this study, we applied translational informatics for intelligent medicine of acute myeloid leukemia, a type of cancer characterized by disease relapses even after seemingly successful treatments. Treatment failure is associated, at least in part, with the fact that targeting individual proteins often promotes rewiring of relevant networks and re-organization of interactions of, among others, non-targeted proteins to eventually evade single-target therapies. To develop efficient therapies, these dynamics should be taken into account and target whole network modules instead of singleton genes in order to prevent the establishment of compensating signaling circuits. Therefore, we integrated network-based methods, structural pharmacology, and molecular modeling to establish two complementary multitargeting strategies, one in the form of repurposable drug combinations and the other as a de novo synthesized triple-targeting agent. Of note, our study exploits, for the first time, a greedy algorithm to identify optimal combinations of drugs and therapeutic protein targets.

## 1. Introduction

Acute myeloid leukemia (AML) is a complex and heterogeneous blood malignancy in adults. Its management includes various therapeutic strategies, mainly intensive chemotherapy, targeted therapies [1,2], allogeneic stem cell transplantation [3], and CAR T-cell immunotherapy [4]. One major clinical challenge of AML is that patients are at a life-long risk for disease relapses even after seemingly successful therapies [5], a condition known as minimal residual disease (MRD). Even when the majority of malignant cells are eliminated by treatments, a few cells manage to survive in the patient long after the initial diagnosis [6] and, under the appropriate conditions, may fuel recurrence and/or resistance to therapies [7,8,9]. Furthermore, selection of best possible care is often complicated by polypharmacy, as these patients may need to receive multiple drugs (often ≥5) in the context of additional lines of cancer therapy, management of comorbidities, and/or supportive care, which generally increases the chances for negative drug–drug interactions [10].

The tendency of AML to recur reflects the dynamic and evolving nature of cancer [11]. Medical treatments exert immense evolutionary pressure for positive selection of drug-resistant clones [12]. Besides genomic alterations, rapid and dynamic rewiring of signalling pathways and transcriptional networks is also a means for developing resistance to therapies [13]. In particular, therapeutic gene targets are usually components of networks that secure their robustness through feedback and redundancy mechanisms. Therapy-induced perturbations of individual gene targets often promote rewiring of relevant networks and re-organization of interactions among other, non-targeted genes, which favor adaptation and survival. Eventually, the signaling circuits that are established under the selective pressure of a certain drug orchestrate the evolution of correspondingly resistant tumor subclones [14]. This realization has shifted the research interest from the `one target-one drug-one disease` paradigm to the identification of compounds that interact with multiple interrelated targets in the cancer network to create an additive or synergistic effect [15]. Hence, next-generation therapies should rely on drugs that do not interact with a single selected hub but rather annihilate the whole network one-off by interfering, at first instance, with as many of its components as possible. Multi-targeting is pursued by either combination therapies, whereby two or more drugs acting on distinct targets are combined [16,17], or multi-target drug design, whereby a single agent acts on two or more interrelated oncogenic targets simultaneously [18]. Both strategies have shown promising efficacy and safety profiles in the AML clinical and/or preclinical setting [19,20,21,22,23,24,25,26].

The identification of suitable proteins and corresponding drugs for multi-targeting can be guided by network pharmacology [27,28]. In this regard, the reconstruction of the core disease networks facilitates the rational selection of the components that should be optimally targeted to inhibit malignant phenotypes while minimizing risks for drug resistance and side effects [29,30,31,32,33]. These approaches do not only identify proteins that can be targeted by already existing compounds but also effectively map those for which there are no clinically available therapeutics, thereby providing a comprehensive means to explore and extend the druggable space of proteins implicated in complex diseases [34,35,36].

Herein, we established a network-based, computer-aided approach for the strategic design of multi-targeting drug combinations and de novo agents that interact with components of the AML network. First, we identified targets overexpressed in AML versus healthy blood cells and exploited them to reconstruct the core AML network. Then, aiming to demolish the AML network one-off and, at the same time, avoid polypharmacy-related complications, we sought to predict the maximum number of targets that are inhibited using as few compounds as possible. We provide robust computational evidence that, on the one hand, the druggable components of the network are potentially targeted by specific combinations of already existing drugs, and on the other hand, key undruggable components of the network can be inhibited simultaneously with a novel small-molecule compound, which we generated in silico using structure-based de novo multi-target drug design. By integrating network-based methods, functional enrichment analysis, phylogenomics, structural pharmacology, and molecular modeling, we determined two alternative strategies with the potential to specifically demolish the AML network, one in the form of drug combinations and the other as a de novo synthesized multi-targeting agent.

## 2. Materials and Methods

### 2.1. In Silico Reconstruction of the AML Network and İdentification of Druggable and Undruggable Hubs

The differentially expressed transcripts in AML patient tissue samples as compared to healthy ones were defined as previously described [8] (Table S1). The locus type of these genes (i.e., protein-coding genes, non-coding etc.) was identified via HGNC BioMart (https://www.genenames.org/, accessed on 11 February 2022) [37]. Potential protein–-protein interactions of the upregulated protein-coding genes (PCGs) were investigated by STRING version 11.5 [38], a database of experimentally verified and predicted physical and functional protein–protein associations derived from diverse sources (e.g., experimental studies, databases, scientific literature mining, etc.); a confidence score of 0.85 was applied so as to avoid false positives and false negatives. The topological features (e.g., degree distribution) of the constructed network were investigated with Cytoscape (v.3.9.0), a software for network processing and visualization. [39]. A greedy algorithm was applied to select the maximum number of the most highly connected nodes in the generated graph being targeted by the minimum number of known drugs. For this purpose, the Python programming language, as well as the Python libraries *pandas* and *NetworkX*, were used for data manipulation and graph analysis, respectively. The corresponding UniProt identifiers (UniProt IDs) for all PCGs were acquired using custom R scripts. The main repository of protein sequences and functional information, UniProt Knowledgebase (UniProtKB) [40] (https://www.uniprot.org/help/uniprotkb, accessed on 25 February 2022), *release 2022_05*, was searched with in-house Python scripts, utilizing the *urllib* and *BeautifulSoup* libraries, to assign UniProt IDs to the PCGs under study. The drugs potentially targeting the PCGs were retrieved from DrugBank (https://go.drugbank.com/, accessed on 25 July 2022) (Table S2). Gene set enrichment analysis (GSEA) [41] was performed to identify relevant statistically significant gene ontology (GO) terms over-represented in those genes encoding the protein components of the constructed AML network (Table S3).

### 2.2. Molecular Dynamics Simulations of DNMT3A in Procaine Environment

To perform the molecular dynamics simulations, we took into consideration that the methyl group donor of DNMTs is S-adenosyl-L-methionine (SAM), which, following transfer of the methyl group onto the DNA, converts into S-adenosyl-L-homocysteine (SAH) [42]. The methylation coproduct SAH usually acts as a competitive inhibitor of DNMTs, and the strength of its binding to DNMTs is often even higher than that of the SAM cofactor itself [42]. Therefore, two atomistic simulations were prepared: (1) SAH-free DNMT3A “apoenzyme” bound to procaine at its SAH-pocket, and (2) SAH-bound enzyme in excess (0.07 M) procaine solution. The atomic coordinates of the enzyme were obtained from the Research Collaboratory Structural Bioinformatics (RCSB) Protein Data Bank (PDB) (PDB ID: 6F57) [43]. The human metabolome database (HMDB ID: HMDB0014859) was used to get the atomic coordinates of procaine. CGENFF4 [44] was used to obtain the forcefield parameters for procaine and SAH. The structures were solvated in a cubic water box with a minimum 12 Å buffer zone between the biological material and the system. Na^+^ and Cl^−^ ions were randomly positioned in each water box to mimic a physiological salt concentration of 0.161 M. The system was neutralized electrostatically with additional Na^+^ or Cl^−^ counterions to balance the net excess charge. For the simulations containing procaine, randomly distributed procaine molecules were introduced to a concentration of 0.07 M. The CHARMM36m force field [45,46], the four-site OPC water model [47], and the most recently updated Na^+^ and Cl^−^ ion parameters were used to describe the interactions between the atoms and chemical groups [48,49]. The molecular dynamics trajectories were collected using GROMACS, version 2019. 4 [50]. Each system box was energy-minimized using a combination of the conjugate gradient and steepest descent algorithms, followed by equilibration in the NVT ensemble for 1 ns at 100 K and 1 ns at 300 K and also in the NVT ensemble using an integration timestep of 1 fs for each. Production trajectories for each of the four enzyme systems were gathered in the NPT ensemble at 300 K and atmospheric pressure (1 atm) using an integration timestep of 2 fs for a total of 500 ns. The temperature and pressure were kept constant using the velocity-rescaling thermostat [51] and the Parrinello–Rahman barostat [52]. The atomic coordinates were recorded every 100 ps. Visual Molecular Dynamics (VMD), version 1.9.4 [53] and its Python wrapper library, VMD Python version 3.0.1, were used for all analyses. Each of the two 500 ns atomistic simulations is replicated three times for the statistical confidence of the results. Further simulation details are provided in Table S4.

### 2.3. Structure-Based De Novo Design of a Triple-Targeting Agent

The de novo design and in silico evaluation of an agent that simultaneously interacts with CCNA1, CCND2, and CCNE1 was performed through the following steps: (1) acquisition of three-dimensional (3D) structures of the cyclin proteins; (2) multi-targeting ligand building; and (3) computation of physicochemical, pharmacokinetic, drug-like, and toxicity parameters.

#### 2.3.1. Acquisition of 3D Structures of the Cyclin Proteins

The experimentally resolved 3D structure of the Homo sapiens CCNE1 was obtained from the Research Collaboratory for Structural Bioinformatics (RCSB) Protein Data Bank (PDB), San Francisco, CA, USA [54]; PDB ID:1W98. ColabFold (https://github.com/sokrypton/ColabFold, accessed on 27 January 2023), which is based on AlphaFold [55], was used to predict the 3D structures of the human CCNA1 (RefSeq ID: NP_003905.1) and CCND2 (RefSeq ID: NP_001750.1) protein sequences.

#### 2.3.2. Multi-Targeting Ligand Building

Ligands were generated based on the 3D structure of the target cyclin proteins by employing LigBuilder V3. This is the first de novo multi-target drug design program, which can be used to design ligands to target multiple receptors, multiple binding sites of one receptor, or various conformations of one receptor. It can be especially applied for the design of common ligands against protein targets with large differences in binding sites [56]. First, we detected potential binding sites in the target cyclins via the ‘Cavity’ module, which considers the structural constraints, hydrophobic effect, and hydrogen bonds. A probe sphere is used to explore the entire surface area of the three cyclins for binding pockets. A pharmacophore model was derived from the target proteins to define key interaction sites, and the druggability of the detected binding sites was estimated. We used the ‘de novo’ design strategy of LigBuilder V3, whereby a sp^3^ carbon with four hydrogen atoms is placed randomly into the binding pocket and provides the starting point upon which a new molecule is built in a stepwise manner by applying local energy minimization at each step. Hence, this workflow does not require a user-defined “seed” structure to be pre-placed into the binding site of the target protein. A build-in genetic algorithm for ligand construction was applied. Initially, organic fragments (i.e., building blocks) were selected from the default fragment library and attached to the seed structure with a synchronous growing operation, toward generating a larger compound that would fit into the active sites of the three target proteins. The new generation of compounds, derived from the parent population, were randomly split into fragments and were recombined—a process called crossover—to form the new “seed structure pool”. The nascent fragments (progeny) are statistically more fit (i.e., privileged) than their parents and are subsequently chosen to serve as “seeds” in the subsequent cycles of ligand design. The overall procedure was reiterated until convergence, that is, the full generation of novel optimal ligand molecules.

ChemDraw (https://revvitysignals.com/products/research/chemdraw, accessed on 22 June 2023) and Chemaxon (https://chemaxon.com/, accessed on 22 June 2023) naming toolkit were used for generating the chemical structure of the ligand and naming it.

#### 2.3.3. Computation of Physicochemical, Pharmacokinetic, Drug-likeness, Synthesizability, and Toxicity Parameters

The drug-like properties of the generated ligand compound were evaluated using the SwissADME (https://www.swissadme.ch/, accessed on 11 July 2023) [57], a freely accessible web tool, which includes a comprehensive set of pharmacokinetic and drug-likeness predictive models. The freely available online tool pkCSM [58], utilizing graph-based structure signatures, was used to assess the toxicity risk of the compound.

### 2.4. Identification of the Amino Acid Residues Mediating the Multi-Target Agent-Cyclin Protein İnteractions

To identify the amino acids that are structurally and functionally important for the interaction between the de novo designed multi-targeting agent and cyclin proteins, the following steps were executed: (1) molecular docking; (2) orthologs search and protein sequence alignments; and (3) creation of amino-acid sequence motifs.

#### 2.4.1. Molecular Docking

The multi-target ligand generated by de novo drug design was validated by performing molecular docking simulations via AutoDock Vina [59]. The ligand was docked against each cyclin protein, and its binding affinity towards cyclins was estimated. The spacing value was set as 0.375 Å by default. Also, the x center, y center, and z center were set as 18.774, −0.488, −20.234; 18.768, −0.557, −20.365; 18.545, −0.404, −20.191 for CCNE1, CCNA1, CCND2, respectively. The van der Waals surface of the ligand–protein binding site was calculated, and the van der Waals surface of the ligand was expanded until it collided with that of its cognate protein.

#### 2.4.2. Orthologs Search and Protein Sequence Alignments

In order to retrieve the orthologous amino acid sequences of the proteins CCNA1, CCND2, and CCNE1 across diverse taxa, the corresponding *Homo sapiens* (Human) and *Mus musculus* (Mouse) protein sequences were used as probes to search the well-annotated genomes of the vertebrate species *Macaca mulatta*, *Equus caballus*, *Monodelphis domestica*, *Ornithorhynchus anatinus*, *Gallus gallus*, *Anolis carolinensis*, *Xenopus tropicalis* and *Danio rerio* in the publicly accessible non-redundant sequence database National Center for Biotechnology Information (NCBI)’s RefSeq [60] by applying reciprocal best hit BLASTp. The orthologous amino acid sequences were aligned with PRALINE [61]. The resulting multiple sequence alignments were edited with Jalview [62].

#### 2.4.3. Creation of Amino Acid Sequence Motifs

The human protein sequences CCNA1, CCND2, and CCNE1 were submitted to the NCBI’s Conserved Domains Database (CDD) [63], which relies on position-specific scoring matrices to identify protein domains and functionally important residues. The sequence motifs harbouring the residues implicated in the ligand–protein interactions were excised from the multiple sequence alignments and were subsequently submitted to WebLogo3 [64], with default options, to construct consensus sequences.

### 2.5. Molecular Visualization

The molecular images were generated using PyMOL Molecular Graphics System, version 2.5.4, Schrodinger LLC. (Hyderabad, Telangana). PoseView was also utilized for the two-dimensional (2D) display of protein–ligand interactions [65].

## 3. Results

### 3.1. A Transcriptomics-Based Reconstruction of the AML Network Drives the Selection of Actionable Targets and Corresponding Multi-Targeting Strategies

**Figure 1:**
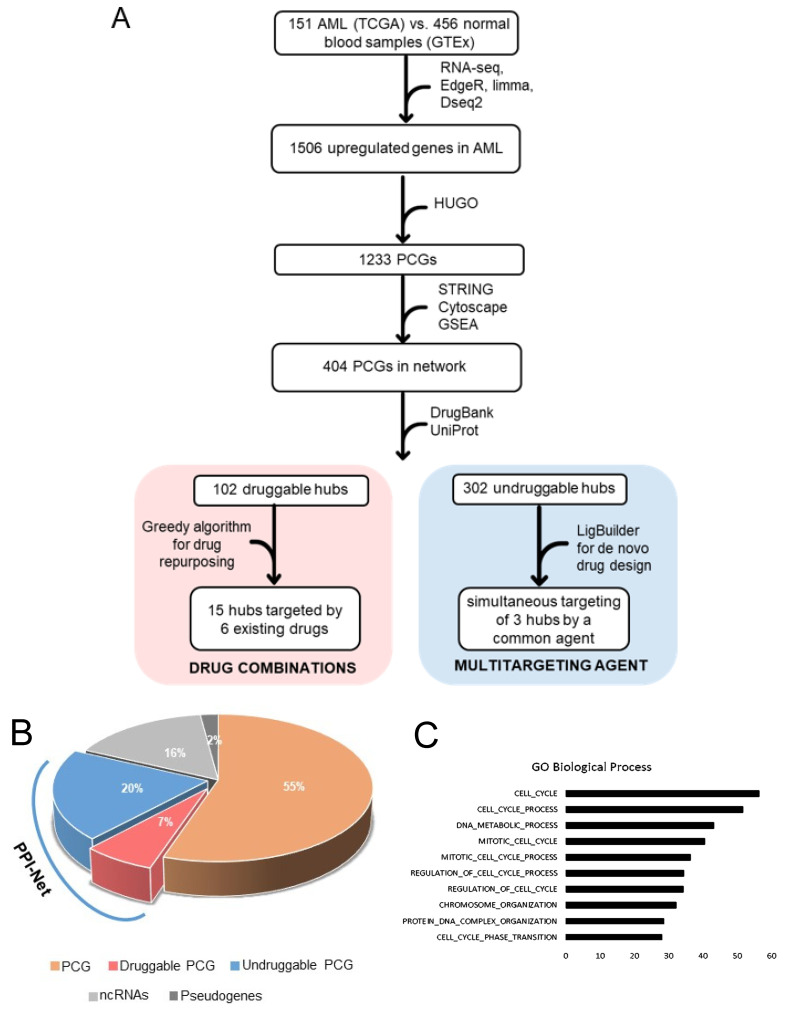

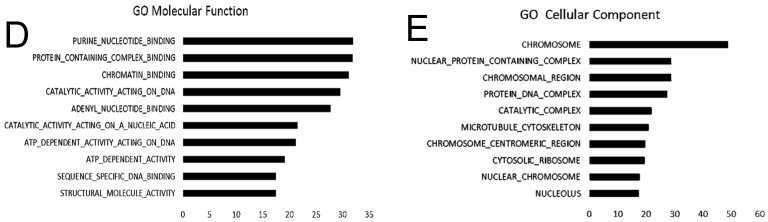
Reconstruction of an AML-specific network drives selection of suitable combinations of targets and appropriate strategies for their simultaneous inhibition. (**A**) The workflow for target/drug selection is based on the topological, functional, and pharmacological properties of the network. (**B**) Overview of the upregulated transcripts in AML cells versus normal blood controls. Of those, 404 transcripts encode proteins that are involved in the AML network. (**C**–**E**) GSEA on the biological processes (**C**), molecular functions (**D**), and cellular components (**E**) that are associated with the 405 transcripts, the products of which participate in the AML network.

To select the suitable combinations of targets, we sought to reconstruct a core AML network, characterize its associated functions, and define its topological and pharmacological properties. This information enables us to prioritize the most efficient combinations of targets and to decide on which multi-targeting strategy would be optimal for their simultaneous inhibition, according to their ability to interact or not with already existing drugs. To this end, we set up the workflow depicted in Figure 1A. As a first step, we compared the gene expression profiles from 151 AML samples available in the TCGA database versus the 456 normal blood samples available in GTEx to identify the differentially expressed genes (DEGs) in leukemic cells versus normal control samples. To reduce heterogeneity introduced by different RNA sequencing analysis packages, we created the consensus list of the DEGs that are identified as commonly up- or down-regulated by three methods, namely edgeR, limma, and DESeq2 [8]. We found 1506 genes that are significantly upregulated in AML versus normal blood (Table S1). By using HGNC BioMart, we further identified the type of genes to which the 1506 upregulated transcripts correspond, such as protein-coding, pseudogene, and non-coding RNA (long non-coding RNA, microRNA, small nucleolar RNA, and small nuclear RNA). Overall, 1233 DEGs were annotated as PCGs, 239 as ncRNA genes, 31 as pseudogenes, and 3 as others (Figure 1B).

To investigate which of the products of the 1233 upregulated PCGs interact physically and/or functionally to make up the core AML network, we analyzed them in the STRING database. We found that 404 PCGs (Table S1) form PPIs and create a highly interconnected network, hereafter termed the ‘AML network’. Gene Set Enrichment Analysis (GSEA) (Figure 1C–E) showed that these components are strongly associated with cell cycle, mitotic and DNA metabolic processes, and chromosome organization. They are also over-represented in molecular functions within the nucleus, such as DNA binding, ATP-dependent and catalytic activities on DNA, and binding on chromatin and protein-containing complexes. These results confirmed that proteins involved in cell cycle control and act on DNA are key components of the AML network and have a prominent role in AML pathogenesis [66,67].

**Figure 2:**
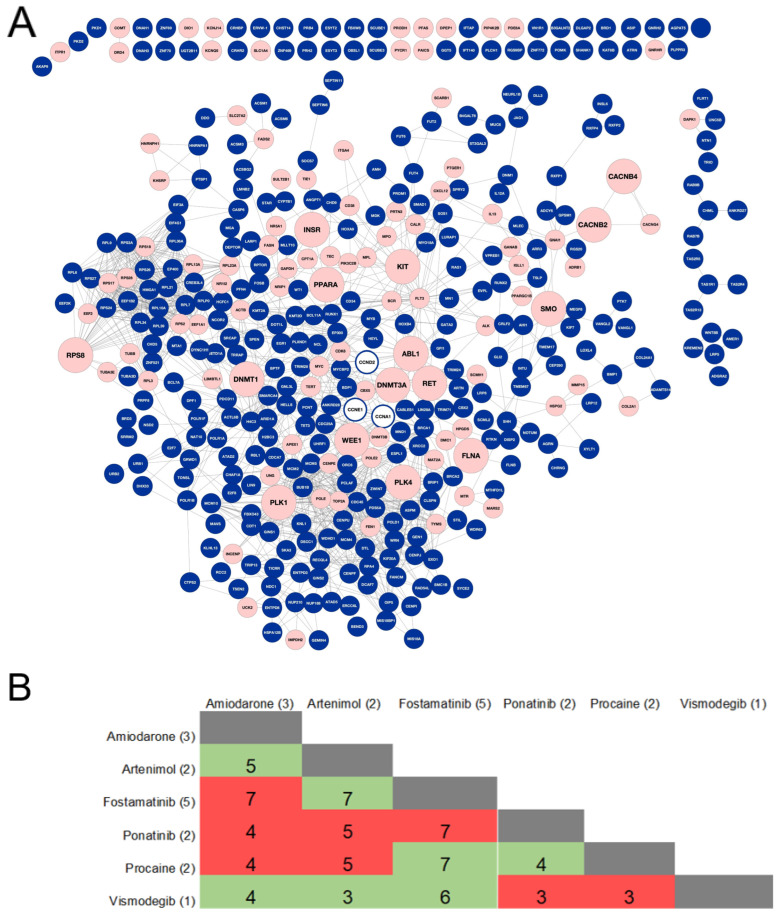
Overview of the druggable (light red) versus undruggable (blue) components of the AML network. (**A**) The nodes represent proteins, and the connecting lines (edges) indicate functional associations. The fifteen selected target proteins are represented by bigger nodes. The three cyclin proteins that were selected for subsequent de novo multi-target drug design are denoted by a blue outline. (**B**) Diagram of the permissible two-drug combinations that can target the highest number of druggable hubs. The safe combinations appear in green, while those that are associated with drug–drug interactions are in red. The number of the AML network hubs targeted by each drug alone is depicted next to each drug name. The number of the targeted hubs for each drug combination is outlined in the corresponding cell.

In the reconstructed network (Figure 2A), the most highly interconnected nodes, corresponding to hubs, are more relevant to the overall function and integrity of the network. Intra-modular hubs are central and have the highest number of connections to the neighboring nodes, whereas inter-modular hubs are intermediate between two or more modules [68,69,70]. These hubs are highly significant for network integrity; hence, their pharmacological inhibition stands higher chances for demolishing the AML network one-off. To decipher such hubs in the AML network, we performed topological analysis via Cytoscape. Subsequently, we classified the network hubs based on their ability to be targeted by existing compounds (Figure 2A). To this end, the corresponding UniProt IDs for the 404 PCGs were acquired from the UniProt Knowledgebase [40] and juxtaposed with the DrugBank data to retrieve the list of drugs potentially targeting the corresponding proteins. We found that 102 nodes in the AML network (Figure 1B and Figure 2A, red-colored nodes) are targeted by 607 unique drugs (Table S2). Therefore, multi-targeting for these proteins can capitalize on the prediction of appropriate combinations of existing drugs. In contrast, 302 nodes in the AML network were defined as ‘undruggable’ (Figure 1B and Figure 2A, blue-colored nodes); hence, de novo drug design is essential to achieve their multi-targeting.

### 3.2. A Greedy Algorithm Predicts Safe Combinations of Approved Drugs for Multi-Targeting of the Hubs in the AML Network

To predict combinations of existing drugs that can demolish the AML network by simultaneously interfering with as many druggable proteins as possible, we exploited a greedy algorithm. This algorithm iteratively selects drugs from the available pool to maximize the cumulative number of targeted proteins. A custom scoring function was introduced for each protein in the network, serving as a pivotal component. This scoring mechanism allowed for ranking the top-15 proteins that could strike an optimal balance between high connectivity within the network and the efficient targeting of proteins by a minimal number of drugs. For this purpose, the Python programming language, as well as the Python libraries *pandas* and *NetworkX* were used for data manipulation and graph analysis, respectively. The algorithm defined 15 hubs, which are the pharmacological targets of the overall 6 existing drugs (Table 1). These are (a) procaine, that targets the DNA methyltransferases DNMT1 and DNMT3A [71]; (b) amiodarone, an agonist of PPARA and inhibitor of the Voltage gated L-type calcium channel proteins CACNB2 and CACNB4; (c) vismodegib, a well-known competitive antagonist of the SMO (smoothened) G protein-coupled receptor, which is a component of the Hedgehog signaling pathway [72]; (d) fostamatinib, a tyrosine kinase inhibitor targets INSR, WEE1, KIT, and PLK1/4; (e) artenimol, a derivative of artemisinin, that targets FLNA and RPS8and (f) ponatinib, a multi-targeted kinase inhibitor, that targets ABL1 and RET. Interestingly, the identified drugs have shown antileukemic activity in AML cell lines, patient samples, or clinical patients. Furthermore, the corresponding hubs inhibited by these drugs have shown potential as therapeutic targets of leukemia based on a number of preclinical and clinical studies (Table 1).

To evaluate combinations of 2 or 3 of these drugs with a favorable safety profile, we used DrugBank’s drug–drug interaction checker, which allows for up to 5 drugs at a time to be checked against one another for potential drug–drug interactions and assigns a relative severity score at culprit pairs. The permissible combinations of 2 drugs appear in Figure 2B, highlighted in green, while the ones posing a risk for drug–drug interactions and clinical complications are depicted in red. In detail, there are 7 combinations free from reportable interactions. Three of them entail vismodegib, which can be safely used with either fostamatinib, amiodarone, or artenimol (multi-targeting of 6, 4, and 3 hubs, respectively). Moreover, procaine can be used either with fostamatinib (7 hubs) or ponatinib (4 hubs). Other combinations with no reportable interactions are artenimol with either amiodarone or fostamatinib (multi-targeting of 5 and 4 hubs, correspondingly). Among all permissible two-drug combinations, fostamatinib plus procaine, as well as fostamatinib plus artenimol, target up to 7 hubs each. Regarding three-drug combinations, two regimens are predicted to be safe and at the same time target a high number of hubs: vismodegib plus artenimol and either fostamatinib (multi-targeting of 8 hubs) or amiodarone (multi-targeting of 6 hubs).

Overall, our approach provided various options for combinations of existing drugs with the potential to interfere with 3 up to 8 hubs in the AML network. This redundancy offers flexibility for personalizing treatment by administering to each AML patient a two- or three-drug combination that is tailored to their clinical condition to achieve the same therapeutic targeting outcome, that is, to demolish one-off the AML network.

### 3.3. Molecular Dynamics Simulations Infer Inhibition of DNMT3A by Procaine via an Allosteric Mechanism of Pharmacological Action

Two of the predicted drug combinations include procaine, a known inhibitor of the DNA methyltransferase enzymes DNMT1 and DNMT3A, which catalyze DNA methylation, and their activation has oncogenic roles in AML [71]. The currently approved hypomethylating agents, such as two cytidine analogues, decitabine or azacytidine [112], are notably toxic in healthy blood cells due to their non-specific mechanism of action. To address these toxicities, alternative approaches for therapeutic manipulation of DNA methylation revolve around the development of selective inhibitors of DNA methyltransferases [104,113,114]. Hence, we turned our attention to procaine, an epigenetic drug that acts against AML by specifically interfering with DNA methylation.

**Figure 3:**
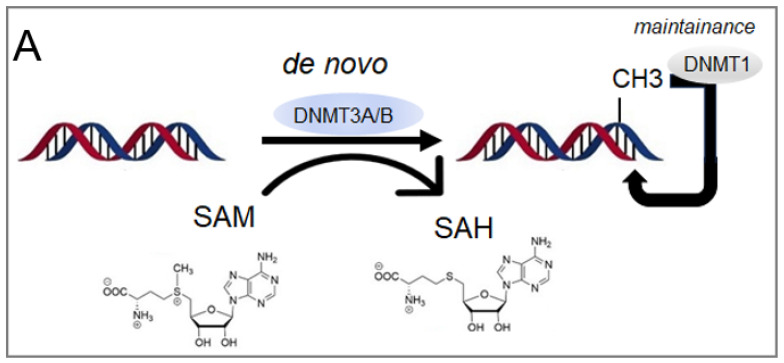

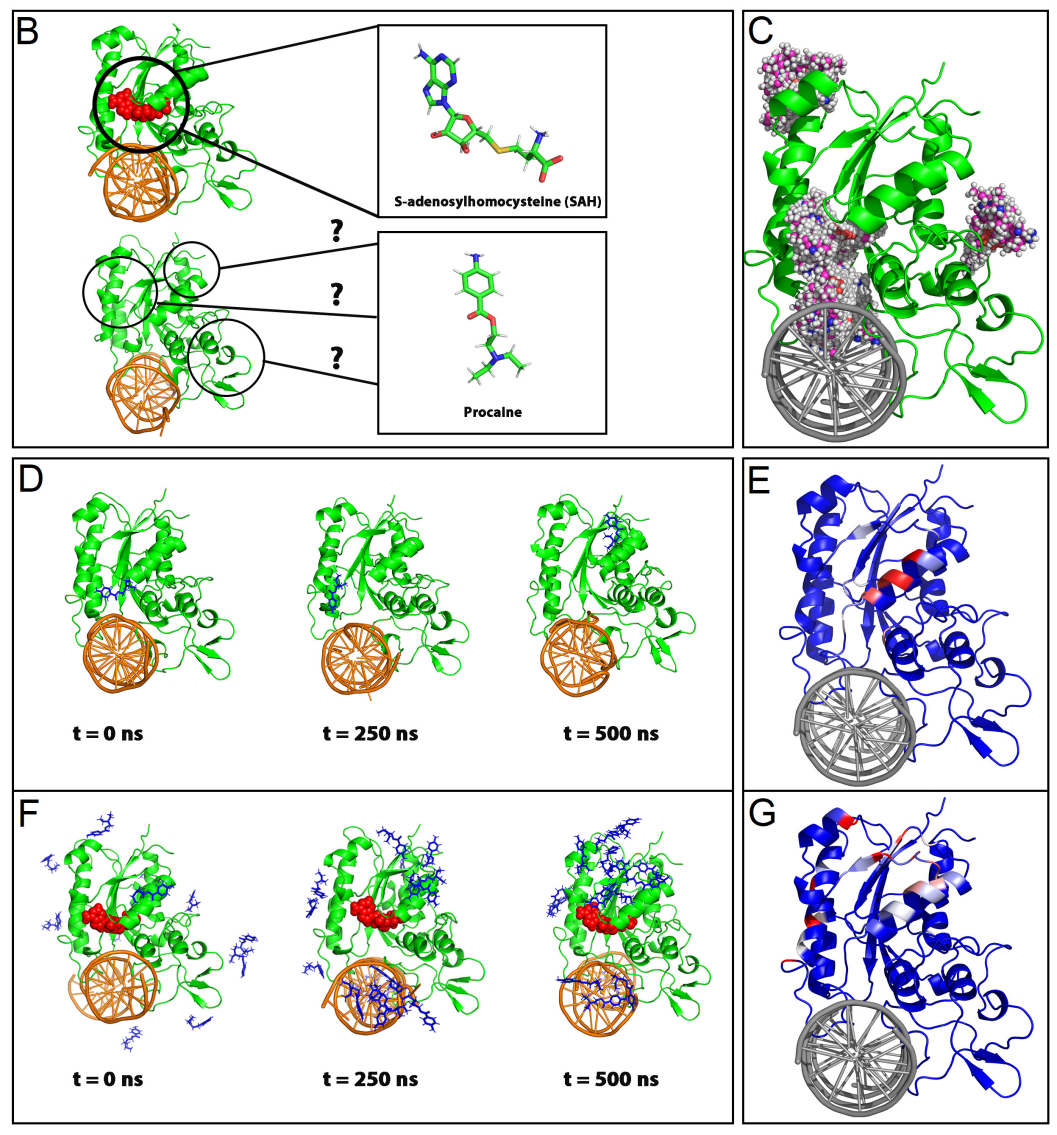
Molecular simulation of the mode of action of procaine on DNMT3A. (**A**) DNMT1, DNMT3A, and DNMT3B are canonical cytosine-5 DNMTs that catalyse the addition of methylation marks to genomic DNA. DNMT3A/DNMT3B are de novo methyltransferases, i.e., they preferentially bind to non-methylated DNA and generate new methylation patterns, while DNMT1 is a maintenance methyltransferase that binds to hemi-methylated DNA during DNA replication and mediates inheritable DNA methylation. The S-Adenosyl-L-homocysteine (SAH) is formed by demethylation of the cofactor S-Adenosyl-L-methionine (SAM). SAH is a potent inhibitor of DNA methylation by selectively binding to the active site of DNMTs, thereby preventing methyl groups from being added to the DNA template. (**B**–**G**) Molecular simulations of DNA-bound DNMT3A in procaine. (**B**) DNMT3A (green) bound to DNA (orange). The cofactor SAH is in red spheres in the top model. The bottom model illustrates the possibilities (indicated by “?”) by which procaine could be interacting with DNMT3A. (**C**) Ensemble of the molecular conformations of procaine as predicted by SwissDock to bind DNMT3A most favorably. (**D**) The snapshots of the active site procaine 1st (ASP1) simulation with initial (time = 0), midpoint (time = 250 ns), and final (time = 500 ns) states from the apoenzyme simulation. DNMT3A (green) bound to DNA (orange). The cofactor SAH is replaced by one copy of procaine (blue), which departs this position at the end of the simulation. (**E**) For the active site procaine 1st (ASP1) simulation, residues that make strong contacts with procaine are indicated in shades of red (red refers to the strongest interaction). (**F**) The snapshots of the excess procaine 1st (EP1) simulation with initial (time = 0), mid-point (time = 250 ns), and final (time = 500 ns) states from the SAH-bound enzyme in 0.07 M procaine. (**G**) Following panel E, strong procaine contacts are indicated for excess procaine 1st (EP1) simulation.

Since the mechanism of pharmacological action of procaine on DNA methyltransferases has not been elucidated, we further performed a detailed analysis of how procaine could interact with its identified targets. DNMT1 and DNMT3A are canonical cytosine-5 DNMT enzymes that catalyze the addition of methylation marks to genomic DNA. DNMT3A acts as de novo methyltransferase, i.e., it preferentially binds to non-methylated DNA and generates new methylation patterns, while DNMT1 is a maintenance methyltransferase that binds to hemi-methylated DNA during DNA replication and sustains inheritable DNA methylation [113,115] (Figure 3A). Therefore, DNMT3A is a particularly crucial target since it functions at the rate limiting step of DNA methylation. For this reason, we focused on the binding dynamics between procaine and functional regions of DNMT3A (Figure 3B, bottom). We exploited ligand docking and atomistic molecular dynamics (MD) simulations to predict how atoms in DNMT3A would move over time in response to procaine. This computational method offers the opportunity to capture the position and motion of every atom at every point in time, which would be otherwise very difficult to address with experimental techniques [116].

To this end, we investigated the binding of procaine to DNMT3A in the presence or absence of SAH by preparing two atomistic simulations: (a) the SAH-free DNMT3A, i.e., apoenzyme, bound to procaine at its crystallographically identified SAH-pocket, and (b) the SAH-bound enzyme (again in its crystallographically identified pocket) in an environment containing excess procaine. The temperature and pressure were kept constant, and the atomic coordinates were recorded every 100 ps. The respective MD simulation trajectories are recorded in Videos S1 and S2. To compare with the MD outcomes, the most favorable binding pockets of procaine on the DNMT3A surface were identified using also unbiased molecular docking [44,117]. The active site of DNMT3A, in close proximity to which SAH is known to reside, was identified as the preferred binding pocket by Swissdock (Figure 3C). This finding suggests a potential interaction between procaine and the crucial catalytic site of the enzyme, which we subsequently tested using MD.

Starting with the active site of DNMT3A occupied by procaine instead of SAH, sub-microsecond active site procaine 1st (ASP1) MD simulation was performed (Figure 3D, left panel, also see Section 2). Contrary to the docking prediction, procaine abandons the SAH pocket (Video S1; Figure 3D, right), relocating to an alternative region on the outer enzyme façade (Figure 3E). Subsequent to the initial simulation, the active site procaine 2nd (ASP2) MD simulation reveals that procaine exhibits some displacement (Figure S1A) but remains close to the SAH-binding pocket, albeit primarily with the help of nonspecific contacts with the DNA (Figure S1B). In the active site procaine 3rd (ASP3) MD simulation replicate, procaine exits the SAH pocket and translocates to a region proximal to the alpha-helix structure in DNMT3A (Figure S1C), eventually stabilizing in a region on the DNA (Figure S1D). The hypothesis derived from these observations suggests that procaine could modulate the activity of DNMT3A allosterically via interacting with distal sites. In order to examine the existence of other allosteric sites on DNMT3A, a second sub-microsecond excess procaine 1st (EP1) simulation was run, in which excess procaine was added to the solvent environment and SAH was reinserted into its binding pocket (Figure 3F). Throughout the entire simulation, SAH remains bound within the active site of DNMT3A, emphasizing its critical role in enzymatic function (Video S2). Procaine molecules map out a small number of distinct loci on the exposed surface of the enzyme, all spatially very distant from the active site (Figure 3G). In the atomistic simulation procedures, steps involving excess procaine 2nd (EP2) and 3rd (ES3) atomistic simulations were performed. Excess procaine molecules were integrated into the solvent environment, while SAH continues to exhibit stable binding within the active site (Figure S1E,G). Individual procaine molecules establish robust interactions at various external sites of DNMT3A, as illustrated in Figure S1F,H. Considering the inhibitory role of procaine in DNMT3A activity, these sites could be implicated in allosteric modulation mechanisms on the protein–ligand interaction landscape of DNMT3A. These MD results indicate that procaine strongly interacts with DNMT3A, albeit at distal sites from its catalytic site, suggesting a potentially allosteric mechanism of inhibition. Furthermore, even at high concentrations, procaine does not preferentially bind to the pocket occupied by SAH, indicating that a competitive binding between SAH and procaine is not likely.

Furthermore, the root mean square deviation (RMSD) (Figure S2A) and root mean square fluctuation (RMSF) metrics (Figure S2B) used in the MD simulation analyses revealed consistently stable conformations for each replicate of the MD simulations of DNA-bound DNMT3A in procaine, in the presence or absence of the cofactor SAH.

### 3.4. De Novo Design of a Novel Multi-Targeting Agent against Undruggable Hubs of the AML Network

A large amount of the hubs of the reconstructed AML network (302 of 404, 74.75%) do not interact with existing drugs. As long as these hubs remain undruggable, they offer opportunities for rewiring to circumvent therapeutic perturbations of the network. Therefore, they represent a source for generating compensatory mechanisms that could support relapse and/or resistance to therapy. Novel compounds need to be developed ‘from scratch’ against these hubs. Therefore, we sought to design a multi-targeting agent that could simultaneously interact with more than 2 of the identified undruggable hubs. According to GSEA analysis, the undruggable hubs are prominently associated with cell cycle processes (Table S3). Based on this finding, it is plausible to envisage that an agent with the potential to interfere with cell cycle regulators that are highly interconnected in the network could secondarily impact many other functionally related hubs, theoretically achieving a ‘domino-effect’ that would lead to the annihilation of the AML network.

**Figure 4:**
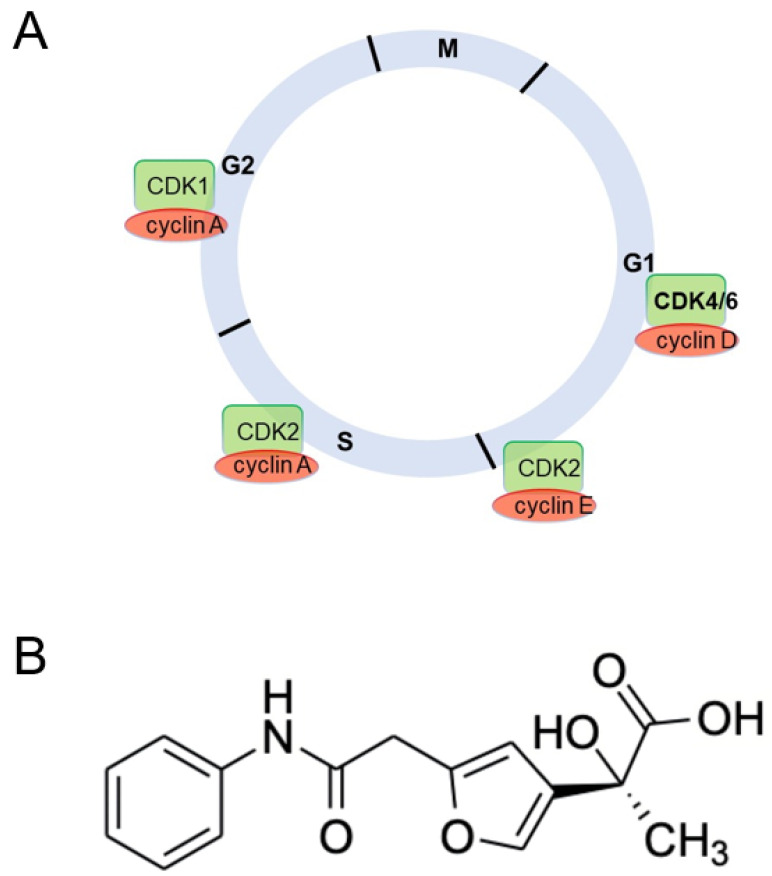
Development of a drug-like ligand to target three cyclins simultaneously. (**A**) Cyclins A1, D2, and E1 are key hubs of the AML network. Complexes of these cyclins with their CDK partners regulate key processes during the cell cycle. CDK4/6/cyclin D complexes act in phase G1, and CDK2/cyclin E complexes act when G1/S transition takes place. The CDK2/cyclin A complex regulates progression through the S phase and the CDK1/cyclin A complex through the G2 phase in preparation for mitosis (M). (**B**) Chemical structure of the triple-targeting cyclin protein ligand that was generated by LigBuilder V3.

Among the undruggable hubs that are over-represented in cell cycle regulation, we spotted three members of the cyclin family, namely cyclins A1, E1, and D2 (CCNA1, CCNE1, and CCND2, correspondingly), which regulate key phases of cell cycle and have been proposed as promising therapeutic targets for haematological cancers (Figure 4A) [118,119,120,121,122]). As CCNA1, CCND2, and CCNE1 are highly interconnected, with 15, 8, and 13 edges correspondingly (Figure 2A), their simultaneous targeting would possibly be propagated across other hubs, eventually impairing the integrity of the AML network. Proteins that belong to the same family have better chances to be targeted by a single compound due to their sequence and structural similarities [18].

We performed structure-based de novo computer-aided drug design for a ligand compound that is able to target all three cyclins. First, we obtained the 3D structures of the human cyclin targets. The structure of the human CCNE1 protein is experimentally resolved and was retrieved from the RCSB PDB [54]. For the structures of human CCNA1 and CCND2, which remain experimentally unresolved, we performed in silico prediction from their primary amino acid sequences using AlphaFold [55]. Then, these structures were used as an input to LigBuilder V3 [56,123]. This software constructs novel chemical compounds by relying on a genetic algorithm that resembles the evolution of a population affected by natural selection. The constructed ligands were subsequently evaluated based on several criteria, including: (a) the lock-key model, which can assess the protein–ligand conformational complementarity; (b) calculation of the ligand–protein binding affinity, using the predicted ligand’s average binding affinity for each target; (c) possession of certain physicochemical features that enable protein–ligand chemical specificity through the receptor binding pocket; and (d) synthesizability of the novel ligand compound [123]. We further assessed the drug-likeness of the ligand by implementing relevant functional modules, such as toxic fragment filtering and Lipinski’s rule of five (RO5) [124], i.e., molecular weight (MW), polar surface area (PSA), rotatable bonds (RB), hydrogen bond acceptors (HBA), hydrogen bond donors (HBD), and LogP.

In this way, (2S)-2-[5-(2-anilino-2-oxoethyl)furan-3-yl]-2-hydroxypropanoic acid with the C_15_H_15_NO_5_ chemical formula and 289.29 g/mol molecular weight was identified as the best scoring drug-like compound targeting CCNA1, CCND2, and CCNE1 among five candidate compounds (Figure S3). Interestingly, only the S enantiomer was identified, while R did not appear in the list of constructed ligands, implying that the interaction with the target proteins is likely stereo-specific. This de novo designed potential multi-targeting agent is hereafter termed a ‘novel ligand’ (Figure 4B).

### 3.5. The Novel Ligand Exhibits Drug-like Features and Lacks Toxicity

To be considered as a drug candidate, a chemical compound should exhibit pharmacokinetic properties including absorption, distribution, metabolism, and excretion (ADME). Moreover, it should have minimal toxicity potential on vital tissues. In this regard, we tested whether the novel ligand exerts essential ADME properties. By employing SwissADME, we found that the novel ligand possesses favorable pharmacokinetic and drug-likeness properties, such as high gastrointestinal absorption, no blood–brain barrier (BBB) permeability or violation of RO5, and a high bioavailability score of 0.56 (56%) (Figure S4A). Furthermore, using the online tool pkCSM [58], we investigated the following toxicity parameters: (a) AMES toxicity, that is an indicator of the mutagenicity of the chemical; (b) the maximum dose of the compound tolerated by humans, where 0.477 log(mg/kg/day) is the threshold toxic dose; (c) minnow toxicity, which represents the concentration of the compound required to cause death of 50% of fathead minnows; a molecule having an LC50 value less than 0.5 mM is considered to be acutely toxic in nature, a skin irritant, and tumorigenic with effect on the reproductive system. Our analysis predicted that the novel ligand is non-mutagenic, non-hepatotoxic, and non-irritant, while the dose range of the compound is considered non-toxic for humans and minnows (Figure S4B), as it would induce toxicity only at elevated doses.

### 3.6. Feasibility of Synthesis of the Novel Ligand

**Scheme 1:**
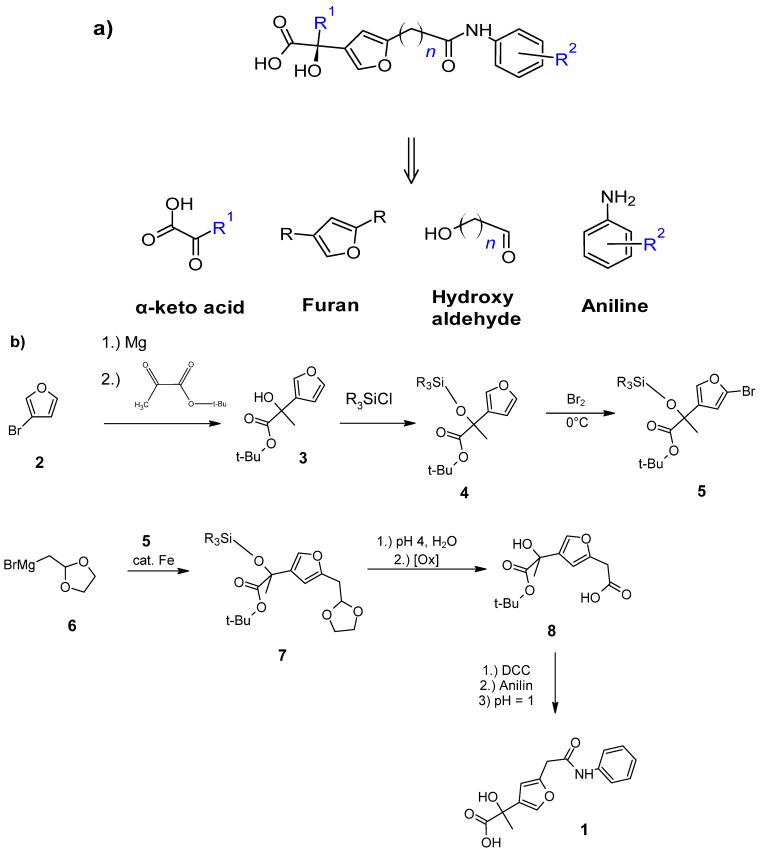
(**a**) Retrosynthetic approach of the ligands. (**b**) Exemplary synthesis of (2S)-2-[5-(2-anilino-2-oxoethyl)furan-3-yl]-2-hydroxypropanoic acid (1), where R1 = CH_3_, n = 1, R2 = H.

The LigBuilder V3 predicted that the novel ligand, namely (2S)-2-[5-(2-anilino-2-oxoethyl)furan-3-yl]-2-hydroxypropanoic acid (1), is synthesizable. Scheme 1a) shows a simple retro synthetic analysis of the cyclin kinase inhibitors. The compound can be in principle prepared from four different building blocks as starting materials, namely furan derivatives, aniline derivatives, hydroxyaldehyde, and α-keto acids. All of these compounds are commercially available with various substituents. To demonstrate this, we provide as an example a synthetic strategy to obtain the lead structure, namely (2S)-2-[5-(2-anilino-2-oxoethyl)furan-3-yl]-2-hydroxypropanoic acid (1), where R1 = Me, n = 1, R2 = H (Scheme 1b).

An effective synthesis of 1 could start with 3-bromo furan and tert-butyl pyruvate; both are compounds that are commercially available or can be prepared by readily available starting materials. It seems feasible that the pyurate could also be replaced with the tert-butyl esters of other α-keto acids, e.g., 2-keto butyric acid, which would allow variability of the alkyl substituent R1. The 3-bromo furan would then first be converted to a Grignard reagent, to which the pyruvate is added, resulting in the tertiary alcohol. Stereoselectivity can be controlled by the addition of titan or copper compounds in combination with chiral ligands [105,106]. For further transformation, the alcohol group of 3 should be protected, for example, in the form of silyl ether (4). It should then be possible to halogenate the furan moiety at the 2 position. The halogenated derivate 5 can be coupled with 2-(bromomethyl)-1,3-dioxolane (6), a commercial reagent that re-ensembles a protected glycolaldehyde. For the C-C coupling between 5 and 6, the latter can be converted to a Grignard reagent using magnesium and added to 5 in the presence of Iron salts as catalysts [125]. If the product 7 is obtained, the acetal could be hydrolysed using dilute acid. This step most likely needs to be carefully optimized to not hydrolyse the tert-butyl ester group, and if desired, we also propose the addition of fluoride ions to deprotect the alcohol group. Successful hydrolysis of the acetal in 7 would then result in a free aldehyde that can be oxidized to the carboxylic acid. Methods for this transformation under mild conditions are already in place [126,127,128,129]. The carboxylic acid 8 could be then converted into 1 by reacting with aniline. Conversion should be feasible under mild conditions using typical coupling reagents like dicyclohexylcarbodiimide and others [130,131,132].

It should be stressed out that, as is the case for any novel chemical compound, the protection strategy of the functional groups is experimentally feasible. It is also needed to be experimentally tested whether the first step of the chemical reaction can be performed in a stereoselective manner. In vivo aspects of the compound, including its half-life in the human body, its administration and metabolic routes, and its pharmacokinetic and pharmacodynamic properties, should also be comprehensively validated in experimental mouse models. Nevertheless, herein we show that retrosynthetic experiments for generating the novel ligand are feasible to start from easily accessible materials.

### 3.7. Molecular Docking Predicts Interaction of the Novel Ligand with Highly Conserved and Functionally Important Residues of the Cyclin Proteins

**Figure 5:**
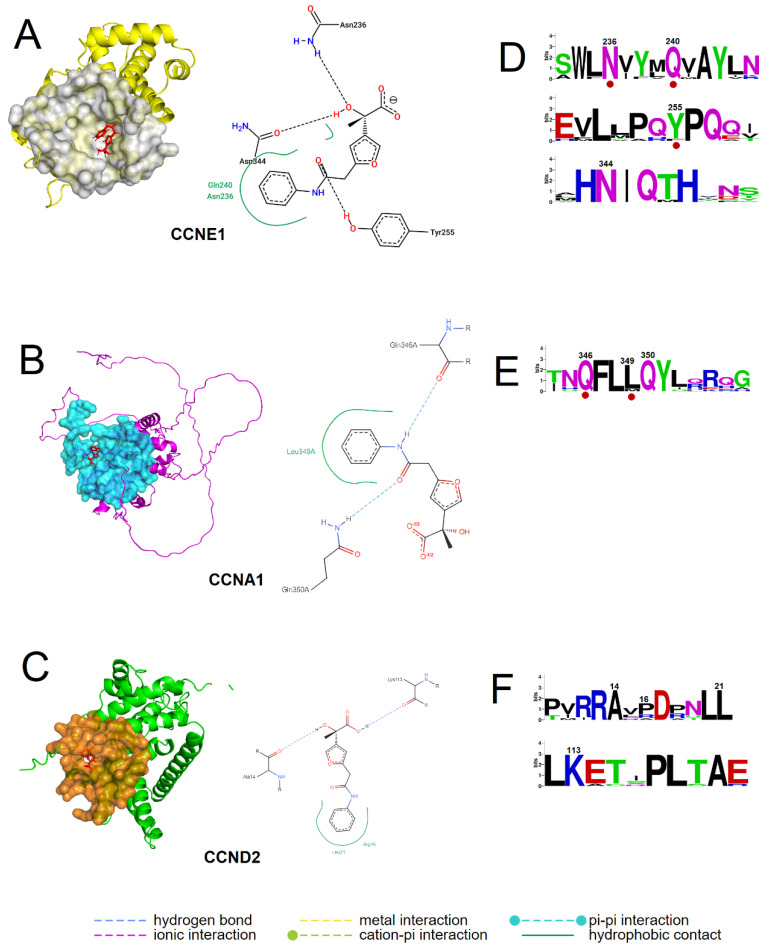
Identification of amino acids involved in the interaction of the novel ligand with cyclin E1, A1, and D2. (**A**–**C**) Docking poses of the ligand against CCNE1, CCNA1, and CCND2. **Left**: Cartoon representation of target proteins and stick representation of the ligand compound. **Right**: 2D view of the protein–ligand binding residues. (**D**–**F**) Logos of corresponding conserved cyclin sequence motifs. The numbers denote the positions of the amino acids that are involved in the ligand–cyclin interaction. The residues reported to reside in the cyclin–CDK interface are indicated by dark red dots. The height of each letter is proportional to the frequency of the occurrence of the corresponding amino acid at that position.

Cyclins have divergent sequences and fulfil diverse functions across the cell machinery. They typically function as cyclin-dependent protein kinase (CDK) activators during cell cycle regulation but may also exert CDK-independent functions [133]. Therefore, we further wondered whether the novel ligand affects regions on the target proteins that are important for their functions, such as amino residues that are involved in the cyclin–CDK interactions. In this regard, we performed molecular docking simulations via AutoDock Vina [59] to map the region(s) of the human CCNE1, CCND2, and CCNA1 where the ligand binds, as well as to estimate the corresponding binding affinities. Given that LigBuilder V3 applies a simple empirical scoring function for calculating the affinities of the ligand–target protein interactions, these simulations serve as an additional line of validation of the novel ligand. The predicted pKd values of the ligand affinity to the proteins CCNE1, CCND2, and CCNA1 are −6.8, −6.2, and −6.4, respectively, indicating that the ligand binds to its target proteins and forms relatively strong interactions with them, in agreement with the LigBuilder predictions. The best docked poses of the ligand compound against cyclins are shown in Figure 5A–C.

The human protein sequences for CCNA1, CCND2, and CCNE1 were submitted to the NCBI’s Conserved Domains Database (CDD) [63] to identify protein domains and functionally important residues based on position-specific score matrices. Moreover, given that functionally important amino acids tend to be conserved across species, we further evaluated the interfaces on which the ligand is predicted to be bound for signs of conservation. To this end, we compared the primary sequences of the human CCNE1, CCND2, and CCNA1 versus the protein products of their orthologous genes in vertebrate species with well-annotated genomes, namely *Macaca mulatta*, *Equus caballus* (Horse), *Monodelphis domestica* (Opossum), *Ornithorhynchus anatinus* (Platypus), *Gallus gallus* (Chicken), *Anolis carolinensis* (Lizard), *Xenopus tropicalis* (Frog), and *Danio rerio* (Zebrafish). Following multiple sequence alignments, the motifs harbouring the residues that mediate the ligand–protein interactions were excised and used to construct consensus sequences (Figure 5D–F). Overall, we found that for CCNE1, the ligand specifically interacts with the amino acids Asn236, Gln240, Tyr255, and Asn344 in the C-terminus of the cyclin-like domain (Figure 5D). These residues are invariant across examined species, whereas Tyr255 is substituted by another highly conserved aromatic amino acid, phenylalanine, in the *Xenopus laevis* sequence (Figure S5). Moreover, Asn236, Gln240, and Tyr255 have a critical role in the CDK–cyclin interface, according to the NCBI CDD database [134], suggesting that these residues could be involved in the cyclin–CDK interaction. The CCNA1 interacts with the ligand through the amino acids Gln346, Leu349, and Gln350, which reside in the C-terminal domain. Of those, Gln346 and Gln350 remain unchanged across species, whereas Leu349 is replaced by the fellow hydrophobic methionine in Zebrafish (Figure 5E and Figure S5). Furthermore, Leu349 and Gln346 were found to reside in the CDK–cyclin interface, suggesting an involvement in the CCNA1–CDK physical interaction (Figure 5E and Figure S5). Regarding the interacting residues in CCND2, the ligand binds to four amino acids in the N-terminal domain. These are three invariant Ala14, Leu21, and Lys113 amino acids, as well as one not conserved residue, Arg16, without any predicted role in the structure or function of CCND2 (Figure 5F and Figure S5).

## 4. Discussion

Recurrence of AML could be prevented by targeting the tendency of networks to establish interactions with alternative hubs in order to circumvent therapeutic perturbations of single targets. In this regard, a paradigm shift is emerging from single-target therapies to the design of multi-target approaches. Simultaneous inhibition of different molecules [15,135,136] is expected to demolish the cancer network one-off and, hence, tackle compensatory network rewiring. Novel multi-targeting agents [18] and drug combinations [137] are the two main strategies for orchestrated pharmacologic targeting and modulation of multiple proteins [136]. Consistently, in this study we established and applied a computational systems biology workflow to identify AML-enhanced proteins that could be inhibited via multi-targeting strategies. Transcriptomics-based reconstruction of the network underlying AML enabled the identification and assessment of targets and drugs with well-balanced profiles between efficacy and safety. Identification of the topological and pharmacological features of the network was followed by the development of distinct multi-targeting strategies, depending on the ability of hubs to be targeted by existing drugs or not. By incorporating diverse computational methods, we developed two parallel sub-pipelines for drug discovery: for druggable hubs, we predicted the optimal combinations of already approved drugs; for rationally selected undruggable hubs (which cannot be targeted by existing drugs), we designed a novel agent that targets all of them simultaneously.

For druggable components, we found that amiodarone, artenimol, fostamatinib, ponatinib, procaine, and vismodegib can be safely combined in 7 dual drug-regimens and 2 triple-drug regimens for multi-targeting perturbation of the AML network. The redundancy of the specific drug combinations offers alternative options for treatment personalization based on the clinical status of each patient. For instance, given that several AML patients may experience neurological implications and neuropathic pain, the predicted procaine-containing combinations could be considered for both, relieving neuropathic symptoms [138], but also for possibly delaying disease recurrence. On a similar note, considering that several AML patients experience treatment-induced cardiovascular diseases [139], the predicted amiodarone-containing regimens may hold promise to exert antileukemic effects while, at the same time, offering prophylaxis from cardiac complications [140]. It is noteworthy that the identified drugs have shown antileukemic activity and repurposing potential for AML in preclinical and/or clinical studies (Table 1), a finding that confirms the prediction accuracy of our established workflow. Furthermore, the use of a greedy algorithm enabled us to minimize the number of these drugs that target a maximum number of hubs to avoid the potential risk for polypharmacy-related complications. To the best of our knowledge, this is the first time to use a greedy algorithm to identify optimal combinations of drugs and therapeutic protein targets. Future experimental testing of these combinations will comparatively estimate their efficacy to annihilate the AML network.

As the mode of action of most of the abovementioned drugs on their identified protein targets is unknown, we got a glimpse into the pharmacological mechanism of procaine against DNMT3A by using a combined approach of docking and MD simulations. The selection of the drug–target pair was particularly motivated by the current intense pharmacological interest for the development of specific DNMT inhibitors in replacement of the currently used but toxic cytidine analogs [112]. We found that the procaine-induced inhibition could occur through allosteric regions of the enzyme rather than a chemical competition with the cofactor SAH. This prediction can be experimentally tested via a double-titration assay including procaine and SAH. Finally, the differences between docking versus MD-predicted binding modalities of procaine are noteworthy. This highlights the importance of the energetic contributions due to the inherent flexibilities of the ligand and the enzyme, as well as the delicate chemical interactions in an explicitly represented solvent environment.

Regarding the undruggable targets, we performed in silico drug design, whereby the newer version 3 of the LigBuilder software was used, combined with protein structure and conservation predictions. This pipeline led to the generation of a novel small-molecule ligand for three different cyclins. The de novo compound appears to exhibit drug-like features, including favorable pharmacokinetic properties and synthesis accessibility, while it is predicted to lack toxicity. Further integration of molecular docking with phylogenetic analyses revealed that the novel agent is likely to bind to distinct, albeit highly conserved and/or functionally important residues in all three cyclin proteins. Whether this ligand holds promise to show antileukemic effects in vitro and in vivo and/or ability to overcome resistance to AML monotherapies remains to be experimentally tested in leukemia cell lines and mouse AML models. Future experiments are anticipated to validate the mechanism of action, test whether the novel agent can efficiently and safely kill leukemic cells through inhibition of cyclins, and subsequently determine whether this agent holds promise to become the first triple-targeting agent against AML.

Overall, we developed two distinct multi-targeting approaches: repurposing of combinations of existing drugs and design of drug-like compounds de novo. Each strategy presents its own advantages and limitations. In particular, drug repurposing expects that existing dossiers of preclinical and clinical information will shortcut expeditious regulatory approval for a new indication, thereby decreasing drug development costs and maximizing clinical utility of the repurposed molecule. However, there are several industrial concerns, such as a clear commercial value proposition, intellectual property, and clinical equipoise, which can complicate the materialization of repurposing for actual clinical use [141]. As far as de novo drug design is concerned, past experience has taught us that generating new compounds from scratch is time-consuming, cost-ineffective, and often linked to high failure rates when forwarded in clinical trials [142]. Nevertheless, artificial intelligence (AI) tools hold promise to generate new chemical entities with desired and customizable features, including aspects of toxicity and pharmacokinetic properties. Computer-aided generation of compounds with a priori designed pharmacological properties may have higher chances for reducing side-effects and/or achieving consistency of preclinical and clinical studies. Overall, if used together, the two strategies could complement each other toward precision medicine for AML. Computational approaches are already streamlining the development of novel therapeutics [142,143]. Synergies of computational methods with experimental testing can markedly improve design–make–test–analyze cycles involved in drug discovery [142], and eventually result in safe multi-targeting approaches that circumvent primary and secondary resistance and improve patient survival.

## 5. Conclusions

In the present study, network pharmacology guided the in silico identification and characterization of targets and drugs with well-balanced profiles between efficacy and safety. An integrative systems biology approach was applied to reposition already approved compounds for potential AML treatment in two- or three-drug combinations. We also discovered a new drug-like compound that potentially targets three AML-relevant cyclin proteins. As cyclins represent attractive drug targets by being the primary regulators of the passage of cells through the cell cycle, this drug-like molecule could be further exploited in future pharmaceutical research endeavors for developing efficient cyclin-targeting drugs.

## Figures and Tables

**Table 1:** Landscape of the role of the identified drugs and their corresponding targets in AML.

Drug	Description-Initial İndication	Protein Target	Effect of Protein Target in IN AML	Drug Repurposing Potential for AML (Type of Data)
Amiodarone	a class III antiarrhythmic for the treatment of recurrent hemodynamically unstable ventricular tachycardia and recurrent ventricular fibrillation.	CACNB2	ND	YES (in vitro) [73].
		CACNB4	CACNB4 downregulates Wnt/β-catenin signalling [74], an important oncogenic signalling pathway that is associated with AML cancer stem cell phenotype [75].	
		PPARA	PPARα increased expression suppresses glucose metabolism and eliminates stem and progenitor cells in AML [76].	
Artenimol	active metabolite of artemisinin and antimalarial agent for the treatment of uncomplicated Plasmodium falciparum infections	FLNA	FLNA regulates rRNA synthesis and cell proliferation in leukemic cells [77]. A variant of KMT2A-FLNA fusion transcript was detected in an AML patient [78].	YES (in vitro) [79,80,81].
		RPS8	RPS8 was identified as one of the proteins differentially expressed between pediatric AML stem cells (AML-SCs) and hematopoietic stem cells [82]. RPS8 protein levels were downregulated in AML-SCs compared to hematopoietic stem cells [82].	
Fostamatinib	tyrosine kinase inhibitor, for the treatment of chronic immune thrombocytopenia after attempting one other treatment.	INSR	INSR codes for the insulin receptor. Insulin promotes the growth of AML blasts and activates the PI3K/Akt and Erk pathways [83]. Downregulated INSR expression may be associated with relapse in AML [84].	YES (in vitro, in patient) [85,86].
		KIT	KIT-D816 mutations are associated with poor prognosis for AML1-ETO-positive AML patients [87].	
		PLK1	Potential therapeutic target in AML. PLK1 is overexpressed in AML cases, and high expression is associated with shortened survival [88]. A novel oral PLK1 inhibitor (onvansertib) has recently been evaluated for relapsed/refractory AML in a phase Ib trial [89]. Onvansertib and decitabine combination is well tolerated and has antileukemic activity particularly in patients with target engagement and decreased mutant circulating tumor DNA (ctDNA) following treatment [89]. Complex karyotype AML was reported to display G2/M signature and hypersensitivity to PLK1 inhibition [90].	
		PLK4	Potential therapeutic target in AML. PLK4 is overexpressed in the TP53-mutant AML subtype and its inhibition induces antileukemic effects [91]. Downregulation of PLK4 induces cell apoptosis and G2/M arrest in AML [92].	
		WEE1	Potential therapeutic target in AML. WEE1 is a mediator of AML cell survival after cytarabine exposure [93]. Ex vivo inhibition of WEE1 and CHK1 synergistically enhanced therapeutic efficacy in AML [94]. Combination of Wee1 inhibitor and cytarabine enhanced anti-leukemic effects in mice with AML [95].	
Ponatinib	kinase inhibitor, for the treatment of various types of CML and Philadelphia chromosome–positive acute lymphoblastic leukaemia	ABL1	BCR-ABL1 fusion transcript is described in an AML case [96]. AML with t(9;22)(q34;q11), also known as AML with BCR-ABL1, is a rare, provisional entity in the WHO 2016 classification and is considered a high-risk disease according to the European LeukemiaNet 2017 risk stratification [97]. ETV6/ABL1 fusion gene has been reported in some AML cases [98]. NUP214-ABL1 fusion gene in an AML patient was detected [99].	YES (clinical trial) Ponatinib showed clinical activity in AML patients with FLT3-ITD in a small phase I study. Regimen optimization and testing in larger cohorts is required [100].
		RET	RET is activated in AML cells; RET-mTORC1 signaling promotes AML through autophagy suppression [101]. ○ Autophagy induction through RET inhibition was proposed as a therapeutic strategy against AML. ○ Caution is needed regarding the therapeutic potential of RET, as autophagy may have opposing roles in oncogenesis, depending on the context. In MLL-AF9 translocated AML models, RET was identified, in vitro and in vivo, to be a potential therapeutic target [102].	
Procaine	local anesthetic, used for peripheral and spinal nerve block	DNMT1	A potentially important target in AML. DNMT1 is 5.3 fold overexpressed in AML cells compared with the control bone marrow cells [103]. A recently characterized DNMT1-specific inhibitor showed better efficacy with improved tolerability against AML in vitro and in vivo [104].	YES (in vitro) [105]
		DNMT3A	A potentially important drug target in AML. Approximately 22% of AML patients harbour DNMT3A mutations affecting translation; these mutations are enriched in intermediate risk cytogenetic profiles [106]. DNMT3A-mutated AML patients have shorter overall survival [106]. Most mutations of DNMT3A are heterozygous affecting the catalytic domain, with R882H mutations being the most frequently observed ones [107]. AML cells carrying the R882H mutation have severely reduced de novo methyltransferase activity and focal hypomethylation at specific CpGs across AML cell genomes [107]. CpG Island hypermethylation mediated by DNMT3A is suggested to be a consequence of AML progression [108].	
Vismodegib	hedgehog pathway inhibitor, for treatment of locally advanced or metastatic basal cell carcinoma	SMO	GLI3R is required for the therapeutic effect of SMO antagonists in AML samples [109]. Component of the Hedgehog signaling pathway, which is implicated in the development, maintenance, and expansion of leukemic stem cells (LSC) chemosensitivity and drug resistance [110].	Equivocal (In a phase Ib clinical trial, vismodegib monotherapy was well-tolerated but had minimal clinical efficacy as a monotherapy in patients who had received prior treatments [111].

## Data Availability

No new data were created or analyzed in this study. Data sharing is not applicable to this article.

## Supplementary Materials

The following supporting information can be downloaded at: https://www.mdpi.com/article/10.3390/cancers16213607/s1, Table S1: AML-associated up-regulated genes and functional analysis of the genes/gene products; Table S2: AML-associated proteins targeted by marketed drugs; Table S3: Gene Ontology analysis of the biological processes associated with the undruggable hubs of the AML network. The hubs over-represented in cell cycle regulation are highlighted in yellow; Table S4: List of three independent molecular dynamics simulations with DNA-bound DNMT3A with varying concentrations of procaine; Figure S1: The independent replicates of 2nd and 3rd molecular simulations of DNA-bound DNMT3A in procaine. (A) The snapshots of the active site procaine 2nd (ASP2) simulation with initial (time = 0), mid-point (time = 250 ns), and final (time = 500 ns) states from the apoenzyme simulation. DNMT3A (green) is bound to DNA (orange). The cofactor SAH is replaced by one copy of procaine (blue), which departs this position at the end of the simulation. (B) For the active site procaine 2nd (ASP2) simulation, residues that make strong contacts with procaine are indicated in shades of red (red refers to the strongest interaction). (C) The snapshots of the active site procaine 3rd (ASP3) simulation with initial (time = 0), mid-point (time = 250 ns), and final (time = 500 ns) states from the apoenzyme simulation. DNMT3A (green) is bound to DNA (orange). The cofactor SAH is replaced by one copy of procaine (blue), which departs this position at the end of the simulation. (D) Following panel B, strong procaine contacts are indicated for active site procaine 3rd (ASP3) simulation. (E) The snapshots of the excess procaine 2nd (EP2) simulation with initial (time = 0), mid-point (time = 250 ns), and final (time = 500 ns) states from the DNMT3A (green) bound to DNA (orange) and SAH (red)-bound enzyme in 0.07 M procaine (blue). (F) Following panel D, strong procaine contacts are indicated for excess procaine 2nd (EP2) simulation. (G) The snapshots of the excess procaine 3rd (EP3) simulation with initial (time = 0), mid-point (time = 250 ns), and final (time = 500 ns) states from the DNMT3A (green) bound to DNA (orange) and SAH (red)-bound enzyme in 0.07 M procaine (blue). (H) Following panel F, strong procaine contacts are indicated for excess procaine 3rd (EP3) simulation; Figure S2: RMSD and RMSF analysis in the MD simulation (A) RMSD analysis on the MD trajectory and (B) RMSF profiles per residue for DNMT3A; Figure S3: The structurally related agents de novo generated with LigBuilder V3; Figure S4: Screenshot of the output of (A) SwissADME and (B) pkCSM; Figure S5: Alignment of the homologous CCNE1, CCNA1, and CCND2 protein sequences. The amino acids are colored based on their conservation scores; Video S1: MD simulation trajectory of the DNMT3A (green) in complex with DNA (orange), with procaine (blue) docked into the SAH binding pocket; Video S2: MD simulation trajectory of the DNMT3A (green) in complex with DNA (orange), in the presence of SAH (red), in a procaine (blue) environment Data.
