# Supplementary material for: Computer-Aided Identification and Design of Ligands for Multi-Targeting Inhibition of a Molecular Acute Myeloid Leukemia Network
Source: Cancers (Basel). 2024 Oct 25;16(21):3607. doi: 10.3390/cancers16213607 (PMC11544916; doi:10.3390/cancers16213607)
Supplement: Supplementary file 1 [file cancers-16-03607-s001.zip › SupplementaryMaterial_20241023/Figure S5.pdf]

# CCNE1

Unconserved 0 1 2 3 4 5 6 7 8 9 10 Conserved

```
                . . . . . . . . . 10 . . . . . . . . . 20 . . . . . . . . . 30 . . . . . . . . . 40 . . . . . . . . . 50
Human_CCNE1      ----------  ----------  ----------  ----------  ----------
Macaca_CCNE1     ----------  ----------  ----------  ----------  ----------
Horse_CCNE1      ----------  ----------  ----------  ----------  ----------
Mouse_Ccne1      ----------  ----------  ----------  ----------  ----------
Platypus_CCNE1   ----------  ----------  ----------  ----------  ----------
Chicken_CCNE1    ----------  ----------  ----------  ----------  ----------
Lizard_CCNE1     ----------  ----------  ----------  ----------  ----------
Opossum_CCNE1    MSRESGSEEY  QEKEEFTQDV  KEEHVDEKED  SNPDELVNGK  EEPSHDELMN
Frog_cyce1       ----------  ----------  ----------  ----------  ----------
Zebrafish_ccne1  ----------  ----------  ----------  ----------  ----------
Consistency      0000000000  0000000000  0000000000  0000000000  0000000000

                . . . . . . . . . 60 . . . . . . . . . 70 . . . . . . . . . 80 . . . . . . . . . 90 . . . . . . . . . 100
Human_CCNE1      ----------  ----------  ----------  ----------  ----------
Macaca_CCNE1     ----------  ----------  ----------  ----------  ----------
Horse_CCNE1      ----------  ----------  ----------  ----------  ----------
Mouse_Ccne1      ----------  ----------  ----------  ----------  ----------
Platypus_CCNE1   ----------  ----------  ----------  ----------  ----------
Chicken_CCNE1    ----------  ----------  ----------  ----------  ----------
Lizard_CCNE1     ----------  ----------  ----------  ----------  ----------
Opossum_CCNE1    GTHEQSHDVP  MNGTHEPSPD  ELFDGEEDPS  IDELVYGDDD  PSFDEVIYEE
Frog_cyce1       ----------  ----------  ----------  ----------  ----------
Zebrafish_ccne1  ----------  ----------  ----------  ----------  ----------
Consistency      0000000000  0000000000  0000000000  0000000000  0000000000

                . . . . . . . . . 110 . . . . . . . . . 120 . . . . . . . . . 130 . . . . . . . . . 140 . . . . . . . . . 150
Human_CCNE1      ----------  ----------  ----------  ----------  ----------
Macaca_CCNE1     ----------  ----------  ----------  ----------  ----------
Horse_CCNE1      ----------  ----------  ----------  ----------  ----------
Mouse_Ccne1      ----------  ----------  ----------  ----------  ----------
Platypus_CCNE1   ----------  ----------  ----------  ----------  ----------
Chicken_CCNE1    ----------  ----------  ----------  ----------  ----------
Lizard_CCNE1     ----------  ----------  ----------  ----------  ----------
Opossum_CCNE1    EDPNPDELVC  EEEGASLDEL  ICEEEDASLD  ELVCEEEGAS  LDELVCEEED
Frog_cyce1       ----------  ----------  ----------  ----------  ----------
Zebrafish_ccne1  ----------  ----------  ----------  ----------  ----------
Consistency      0000000000  0000000000  0000000000  0000000000  0000000000

                . . . . . . . . . 160 . . . . . . . . . 170 . . . . . . . . . 180 . . . . . . . . . 190 . . . . . . . . . 200
Human_CCNE1      ----------  ----------  ----------  ----------  ----------
Macaca_CCNE1     ----------  ----------  ----------  ----------  ----------
Horse_CCNE1      ----------  ----------  ----------  ----------  ----------
Mouse_Ccne1      ----------  ----------  ----------  ----------  ----------
Platypus_CCNE1   ----------  ----------  ----------  ----------  ----------
Chicken_CCNE1    ----------  ----------  ----------  ----------  ----------
Lizard_CCNE1     ----------  ----------  ----------  ----------  ----------
Opossum_CCNE1    PNADELICEE  DPSPDELMCD  EEEASPEELI  CDEEDPHADE  LICEEEDPNP
Frog_cyce1       ----------  ----------  ----------  ----------  ----------
Zebrafish_ccne1  ----------  ----------  ----------  ----------  ----------
Consistency      0000000000  0000000000  0000000000  0000000000  0000000000

                . . . . . . . . . 210 . . . . . . . . . 220 . . . . . . . . . 230 . . . . . . . . . 240 . . . . . . . . . 250
Human_CCNE1      ----------  ----------  ----------  ----------  ----------
Macaca_CCNE1     ----------  ----------  ----------  ----------  ----------
Horse_CCNE1      ----------  ----------  ----------  ----------  ----------
Mouse_Ccne1      ----------  ----------  ----------  ----------  ----------
Platypus_CCNE1   ----------  ----------  ----------  ----------  ----------
Chicken_CCNE1    ----------  ----------  ----------  ----------  ----------
Lizard_CCNE1     ----------  ----------  ----------  ----------  ----------
Opossum_CCNE1    DDLICEEEDP  NVDLICEEEI  LHPDEEDDFI  CEEEDPNPID  LICEEEDPSF
Frog_cyce1       ----------  ----------  ----------  ----------  ----------
```

```
Zebrafish_ccne1 ---------- ---------- ---------- ---------- ----------
Consistency     0000000000 0000000000 0000000000 0000000000 0000000000

                .........260.......270.......280.......290.......300
Human_CCNE1     ---------- ---------- ---------- ---------- ----------
Macaca_CCNE1    ---------- ---------- ---------- ---------- ----------
Horse_CCNE1     ---------- ---------- ---------- ---------- ----------
Mouse_Ccne1     ---------- ---------- ---------- ---------- ----------
Platypus_CCNE1  ---------- ---------- ---------- ---------- ----------
Chicken_CCNE1   ---------- ---------- ---------- ---------- ----------
Lizard_CCNE1    ---------- ---------- ---------- ---------- ----------
Opossum_CCNE1   RELICGEQDP SLHELFDGTD EPRPNRRENP TPDGREDPKP DGREDPNPDN
Frog_cyce1      ---------- ---------- ---------- ---------- ----------
Zebrafish_ccne1 ---------- ---------- ---------- ---------- ----------
Consistency     0000000000 0000000000 0000000000 0000000000 0000000000

                .........310.......320.......330.......340.......350
Human_CCNE1     ---------- ---------- --------MP RERRERD-AK ERDTMKEDG-
Macaca_CCNE1    ---------- ---------- --------MP RERRERD-AK ERDTMKEDG-
Horse_CCNE1     ---------- ---------- --------MP RERRERD-AQ ERDGMKEDS-
Mouse_Ccne1     ---------- ---------- --------MP ---RERD-ST DHSNMKEEG-
Platypus_CCNE1  ---------- ---------- ---------- -MREDTN--- ----------
Chicken_CCNE1   ---------- ---------- ---------- -MRRESDCAE EKAPAKGEG-
Lizard_CCNE1    ---------- ---------- --------MP ---SESDHQD KSAAIHEGG-
Opossum_CCNE1   KQDPSAGELF DGKENPRLNE HADGREERSP DGKEENKKSE TDSGREQRGS
Frog_cyce1      ---------M PVISNPPVEK GTKG-EER-- ---------- ----------
Zebrafish_ccne1 --MPSKKVLQ --------TE HINTTDEA-P ---------- ----------
Consistency     0000000000 0000000000 0000000024 0124534032 2221233230

                .........360.......370.......380.......390.......400
Human_CCNE1     GAEFSARSRK RKANVTVFLQ DPDEEMAKID RTARDQCGSQ -PWDN-NAVC
Macaca_CCNE1    GAEFSARSRK RKANVAVFLQ DPDEEMAKID RTARDQCGSQ -PWDN-NTGC
Horse_CCNE1     SADVSVRSRK RKANVAVFLQ DPDEEIAKMD KTVRSQCGRQ -SWDS-NAVC
Mouse_Ccne1     GSDLSVRSRK RKANVAVFLQ DPDEEIAKID KTVKSEDSSQ -PWDD-NSAC
Platypus_CCNE1  -ADFTGRSRK RKADVAVFLQ DPDEEIAKAD VTKKRQFGNQ -PWNNGDAIY
Chicken_CCNE1   GAEGTVRARK RKADVATFLQ DPDEEIAKIE MSRKKQYENQ LSWNN---IN
Lizard_CCNE1    APDFAVRSRK RKADVVIFLP DPDEDVAGIQ MMKKKQNECQ QPSSN-----
Opossum_CCNE1   GSTDSSRSRK RKADVAVFLL DPDEVLAKRE TIGRT--AVR IP-----AFL
Frog_cyce1      -ASCSVRSRK RKADVAIYLQ DPDETLDCLE MTKKKQYQDR GPWSN-EMIC
Zebrafish_ccne1 -KTTSVRPRK RKADVAIHLQ DPDEEVT--E MTRKKQCASQ ACW-NPDTGY
Consistency     365375*7** ***7*888*7 ****677547 4648572448 0663603333

                .........410.......420.......430.......440.......450
Human_CCNE1     ADPCSLIPTP DKEDD----- DRVYPNSTCK PRIIAPSRGS PLPVLSWANR
Macaca_CCNE1    ADPCSLIPTP DKEDD----- ERVYPNSTCK PRIIAPSRGS PLPVLSWANR
Horse_CCNE1     ENPCSLIPTP DKEED----- ELVYPN--CG PQSIAPSRAS PLPVLNWANR
Mouse_Ccne1     VDPCSFIPTP NKEEDN---- ELEYPRTAFQ PRKIRPPRAS PLPVLNWGNR
Platypus_CCNE1  GNPCALIPTP DKEDDP---- ASPSSPYGLR RFNL--TRTS PLPLLGWANR
Chicken_CCNE1   KDPHMLIPTP DKDDDP---- VGVDYSHFI- HLNVASTRSS PLPILGWANR
Lizard_CCNE1    -LPHTLIPTP DKEE------ -LRSPKFSSI HKSLTAARDS PLPVLGWGNR
Opossum_CCNE1   RNPWSVILTP DKGGD----- EPSLPNSNMP THNFSQHRAT PLPLLGWANR
Frog_cyce1      KSPHKLIPTP EKEEHE---- QNPTSYPHFA SLRFSPVSVS PLPRLGWANQ
Zebrafish_ccne1 TSPCRRIPTP DEVEEPVAFG SVGFTQYASE SIFITPTRST PLPALCWASK
Consistency     35*456*8** 8966610000 4334543332 4337444848 ***6*5*898

                .........460.......470.......480.......490.......500
Human_CCNE1     EEVWKIMLNK EKTYLRDQHF LEQHPLLQPK MRAILLDWLM EVCEVYKLHR
Macaca_CCNE1    EEVWKIMLNK EKTYLRDQHF LEQHPLLQPK MRAILLDWLM EVCEVYKLHR
Horse_CCNE1     EEVWKIMLNK EKTYLRDKHF LQRHPLLQPK MRAVLLDWLM EVCEVYKLHR
Mouse_Ccne1     EEVWRIMLNK EKTYLRDEHF LQRHPLLQAR MRAVLLDWLM EVCEVYKLHR
Platypus_CCNE1  DEVWKIMLNK EQTYLRDKHF LQRHPLLQPK MRAILLDWLM EVCEVYKLHR
Chicken_CCNE1   DDVWKNMINK EETYVRDKLY MQRHPLLQPK MRTILLDWLM EVCEVYKLYR
Lizard_CCNE1    DEVWKNMIKK EQTYVRDKLY MQRHPLLQPK MRVILLDWLM EVCEVYKLHR
Opossum_CCNE1   DEVWKIMLSK EQVYLRDKNF MERHPSLQPR MRSILLDWMM EVSEVYKLHR
Frog_cyce1      DDVWRNMLNK DRTYLRDKNF FQKHPQLQPN MRAILLDWLM EVCEVYKLHR
Zebrafish_ccne1 DEVWNNLLGK DKLYLRDTRV MERHPNLQPK MRAILLDWLM EVCEVYKLHR
Consistency     88**75997* 877*8**647 787**5**87 **79****9* **8*****9*

                .........510.......520.......530.......540.......550
```

```
Human_CCNE1      ETFYLAQDFF DRYMATQENV VKTLLQLIGI SSLFIAAKLE EIYPPKLHQF
Macaca_CCNE1     ETFYLAQDFF DRYMATQENV VKTLLQLIGI SSLFIAAKLE EIYPPKLHQF
Horse_CCNE1      ETFYLAQDFF DRYMATQQNV VKTLLQLIGI SSLFIAAKLE EIYPPKLHQF
Mouse_Ccne1      ETFYLAQDFF DRYMASQHNI IKTLLQLIGI SALFIASKLE EIYPPKLHQF
Platypus_CCNE1   ETFYLAQDFF DRFMATQQNI VKTLLQLIGI SSLFIAAKLE EIYPPKLHQF
Chicken_CCNE1    ETFYLAQDFF DRFMATQQNV VKTLLQLIGI SSLFIAAKLE EIYPPKLHQF
Lizard_CCNE1     ETFYLAQDFF DRYMATHQNV VKTLLQLIGI SSLFIAAKRE EIYPPKLCQF
Opossum_CCNE1    ETYYLAQDFF DRYMATQRNI TKTLLQLIGI TSLFIAAKLE EIYPPKLYQF
Frog_cyce1       ETFYLGQDFF DRFMATQKNV IKSRLQLIGI TFLFIAAKLE EIYPPKLHQF
Zebrafish_ccne1  ETFYLGQDYF DRFMATQENV LKTTLQLIGI SCLFIAAKME EIYPPKVHQF
Consistency      **9**8**9* **8**986*9 7*97****** 86****9*7* ******97**

                 .........560.........570.........580.........590.........600
Human_CCNE1      AYVTDGACSG DEILTMELMI MKALKWRLSP LTIVSWLNVY MQVAYLNDLH
Macaca_CCNE1     AYVTDGACSG DEILTMELMI MKALKWRLSP LTIVSWLNVY MQVAYLNDLH
Horse_CCNE1      AYVTDGACSG AEILTMELII MKALKWRLSP LTIVSWLNVY MQVAYLNDLY
Mouse_Ccne1      AYVTDGACSG DEILTMELMM MKALKWRLSP LTIVSWLNVY VQVAYVNDTG
Platypus_CCNE1   AYVTDGACTE DEILSMELII MKALKWNLSP LTVVSWLNVY MQVAYLNDFY
Chicken_CCNE1    AYVTDGACTE DEILSMELII MKALNWNLNP LTVVSWLNIY MQVAYLNELY
Lizard_CCNE1     AYVTDGACTE EEILNMELLI MKALDWHLSP LTVVSWLNIY LQIAYLNEIY
Opossum_CCNE1    AYVTDGACTE EEILTMELII MKALKWRLSP MTLVSWLNVY MQVAYLNDLY
Frog_cyce1       AFITDGACTE DEITSMELII MKDLDWCLSP MTVVSWFNVF LQVAYIRELQ
Zebrafish_ccne1  AYVTDGACTE DDILSMEIII MKELNWSLSP LTPVAWLNIY MQMAYLKETA
Consistency      *99*****75 79*87**979 **7*6*4*9* 8*7*9*8*99 8*8**87864

                 .........610.........620.........630.........640.........650
Human_CCNE1      -EVLLPQYPQ QIFIQIAELL DLCVLDVDCL EFPYGILAAS ALYHFSSSEL
Macaca_CCNE1     -EVLLPQYPQ QIFIQIAELL DLCVLDVDCL EFPYGILAAS ALYHFSSSEL
Horse_CCNE1      -EVLLPQYPQ QVFIQIAELL DLCVLDVGCL EFSYGVLAAS ALYHFSSSEL
Mouse_Ccne1      -EVLMPQYPQ QVFVQIAELL DLCVLDVGCL EFPYGVLAAS ALYHFSSLEL
Platypus_CCNE1   -EVLVPRYPQ QIFVQITELL DLCVLDVGCL EFPYGVLAAS ALYHFSSSEL
Chicken_CCNE1    -EVLLPQYPQ QIFVQIAELL DLCVLDIGCL EYTYGVLAAS ALYHFSSSEL
Lizard_CCNE1     -EMLLPHYPQ QIFVQIAMLL DLCVLDIGCL GYTYSVLAAS ALYHFSSCEL
Opossum_CCNE1    EEVLMPQYPQ QIFVQVAELL DVCILDMGCF DFTYGVLAAS ALYHFSSTEI
Frog_cyce1       -HFLLPQFPQ EVYIQIVQLL DLCVLDICCL EYPYGVLAAS ALYHFSCPEL
Zebrafish_ccne1  -EVLTAQYPQ ATFVQIAELL DLCILDVRSL EFSYSLLAAS ALFHFSSLEL
Consistency      087*7879** 8899*987** *9*9**8488 785*88**** **9***84*9

                 .........660.........670.........680.........690.........700
Human_CCNE1      MQKVSGYQWC DIENCVKWMV PFAMVIRETG SSKLKHFRGV ADEDAHNIQT
Macaca_CCNE1     TQKVSGYQWC DIENCVKWMV PFAMVIRETG SSKLKHFRGV ADEDAHNIQT
Horse_CCNE1      MQRVSGYQWC DIEKCVKWMV PFAMVIRETG SSKLKHFRGV PAEDAHNIQT
Mouse_Ccne1      MQKVSGYQWC DIEKCVKWMV PFAMVIREMG SSKLKHFRGV PMEDSHNIQT
Platypus_CCNE1   MRKVSGYEWR DVEECVKWMV PFAVAIREAG SSKLKRFRGV PTEDLHNIQT
Chicken_CCNE1    MQKVSGYEWC EIEECVKWMV PFAMAIREVG SSKLKHFRGI APEDLHNIQT
Lizard_CCNE1     MQRVSGYEFS DIEDCVKWMV PFAMVIKEVG STTMKQFRGV PAEDLHNIQT
Opossum_CCNE1    MKKVSGFDWP EVEECVKWMV PFAMAVKEVG GARLKYFKGV PSEDMHNIQT
Frog_cyce1       VEKVSGFKLT ELQGCIKWLV PFAMAIKDGG KSKLKFFKGV DIEDVHNIQM
Zebrafish_ccne1  VIKVSGLKWC DLEECVRWMV PFAMSIREAG SSALKTFKGI AADDMHNIQT
Consistency      768***7674 8895*99*9* ***969895* 7879*4*8*9 539*5****8

                 .........710.........720.........730.........740........
Human_CCNE1      HRDSLDLLDK ARAKKAMLSE QNRASPLPSG LLTPPQSGKK QSSGPEMA
Macaca_CCNE1     HRDSLDLLDK ARAKKAMLSE QNRASPLPSG LLTPPQSSKK QSSEPEMA
Horse_CCNE1      HLNSLDLLDK AQAKKAILSE QNRISPPPTG VLTPPQSSKK QSSGQGTV
Mouse_Ccne1      HTNSLDLLDK AQAKKAILSE QNRISPPPSV VLTPPPSSKK QSSEQETE
Platypus_CCNE1   HVNSLDLLDR AQAKQAILAE HNRTSPLPTG VLTPPQSSKK QTTERKAV
Chicken_CCNE1    HINSLDLLDK AQAKQAILAE QNRTSPFPTG VLTPPQSSKK QPAGLKPI
Lizard_CCNE1     HINTFDLLDK VESKQASLAE KSRHSPLPPG ILTPPQSSKK LPQL----
Opossum_CCNE1    HVNTINLLDR AYEKQAILAD E-KVSA-PSG VLTPPQSDKK PSKEQE--
Frog_cyce1       HTGCLELMEK VHINRALLEE QNRASPIPSG VLTPPQSNKK QKSDPAD-
Zebrafish_ccne1  HVPYLEWLGK VHSYQLVDIE SSQRSPVPTG VLTPPPSSEK PESTIS--
Consistency      *556878978 7567786869 6684*83*68 8****7*79* 65643411
```

# CCNA1

Unconserved 0 1 2 3 4 5 6 7 8 9 10 Conserved

```
               . . . . . . . . . 10 . . . . . . . . . 20 . . . . . . . . . 30 . . . . . . . . . 40 . . . . . . . . . 50
Human_CCNA1     METGFPAIMY PGSFIGGWGE EYLS--WE-- GPGLP----- -DFVFQQQPV
Macaca_CCNA1    METRFPAVMY PGSFIGGWGD EYLC--WE-- RQGLP----- -DFNFQQQRV
Opossum_CCNA1   MNRRDQRARR ----VPSWFG HHLSGRWEEG RRAVPIRCPR KGRDLHVWPG
Mouse_Ccna1     ---------- ---------- ---------- ---------- ----------
Horse_CCNA1     ---------- ---------- ---------- ---------- ----------
Platypus_CCNA1  ---------- ---------- ---------- ---------- ----------
Lizard_CCNA1    ---------- ---------- ---------- ---------- ---MLQSSIA
Chicken_CCNA1   ---------- ---------- ---------- ---------- ----------
Frog_ccna1      ---------- ---------- ---------- ---------- ------MRRS
Zebrafish_ccna1 ---------- ---------- ---------- ---------- ----------
Consistency     0000000000 0000000000 0000000000 0000000000 0000000000

               . . . . . . . . . 60 . . . . . . . . . 70 . . . . . . . . . 80 . . . . . . . . . 90 . . . . . . . . . 100
Human_CCNA1     ESEAMHCSNP KSGVVLATVA RGPDACQILT RAP-LGQDPP -QRTVLGLLT
Macaca_CCNA1    ESEAMHCSNP KNGVVLATVA RGPDACQILT RAP-LGQDPP -QRTVLGLLT
Opossum_CCNA1   ETEAMRRSIS KGRIPLAPAA RSQEICQVVA RAQ-PIPDPP -QRTVLGLLT
Mouse_Ccna1     ----MHRQSS KSGVALPPVG QGPDACQMLS RAQ-LGQDPP -QRTVLGVLT
Horse_CCNA1     ----MHCSSS KSGVVLAPVS RGPDACQMIT RAH-LGQDPP -QRTVLGVLT
Platypus_CCNA1  ----MRRSGS RGRTPQTPVA RPQEIYQVAA RLR-PGQATR -QRTILGLLS
Lizard_CCNA1    VPENMDPSGK DKE--KEQVG RWDNLSEM-A EPH-AHQNAP -QRAVLREIS
Chicken_CCNA1   ---------- --------MR RTGETSQAGR REP-LPAAPR GGRTVLGVLV
Frog_ccna1      MVSGGHILTA SSLVGTGNAF QNQCLAKVEV QSN-LLPPRV SQRTVLGVIS
Zebrafish_ccna1 -------MSS RS---FAPLS SRQE--NILG RAEGLRQLKP GQRIVLGVLT
Consistency     1011432534 5423144465 7345447634 8540536455 08*79*8697

               . . . . . . . . . 110 . . . . . . . . . 120 . . . . . . . . . 130 . . . . . . . . . 140 . . . . . . . . . 150
Human_CCNA1     ANGQYRRTCG QGITRIRCYS GSENAFPPAG KKALPDCGVQ EPPKQGFDIY
Macaca_CCNA1    ENGQYRRTCG QGITRIRCYS GSENAFPPAG KKALPDCGVQ EPPKQGFDIY
Opossum_CCNA1   ENGQYRRTCG QGITTIRCFS GSENVFPPAG KKLISNCMVH VPAKQGFDIY
Mouse_Ccna1     ENEQYRRTCG QEITAIRCFS GSENVFPAAG KKVLSDHGVN EPAKRGFDIY
Horse_CCNA1     ENGQYRRTCG QGITTLRCFS GSENVFPSTG KKLLSDCGVH VPAKQGFDIY
Platypus_CCNA1  ENGQYRKAYG QCITTIRCFS GSENVCPPAG KKSRSNCVVH LPTKQGFSIY
Lizard_CCNA1    ENGQCRRPCV QGMTIVKHLS GAENVFPAAR ESVLSVC--- LPSKQDFDVY
Chicken_CCNA1   ENGQHRLGV- QNTAITRHLS GSENAFPPAG KDEVSNCVVS TTSKQDFAIY
Frog_ccna1      DNDQQKRSVG QGGAPAKCLP GIENVLPFAG KILPTNPALV AP-KPIFTVY
Zebrafish_ccna1 ENEQHNRIFG Q------VAS KYETTLQEVS KRDVST---- ------FRAS
Consistency     8*6*587556 *446356558 86*8668487 9545654252 363864*588

               . . . . . . . . . 160 . . . . . . . . . 170 . . . . . . . . . 180 . . . . . . . . . 190 . . . . . . . . . 200
Human_CCNA1     MDELEQGDRD SCS--VREGM AFEDVYEVDT GTLKSDLHFL LDFNT-----
Macaca_CCNA1    MDELEQGDGD SCS--GREGM AFEDVYEVDT STLKSDLHFL LDFNT-----
Opossum_CCNA1   VDEPEHGERC SCSCTVREEI AYEDVYEVDT STLKSDLHFL LDFST-----
Mouse_Ccna1     MDDPEQGDRD TCS--GKEGI IFEDVYEVDT SMLKSDLHFL LDFNT-----
Horse_CCNA1     MDEPEQGDRD SCT--EREGM AFEDAYEVDT STLKSDLHFL LDFST-----
Platypus_CCNA1  IDKPERGERR GCGCTVGEQV EYGDVREAES KAAKPDLHFL LDFSTGNICC
Lizard_CCNA1    VDDVE--ERS WCSYMAVEEV E-PTMCEIDS SALKPNIHLL LTLSTG----
Chicken_CCNA1   IDEPEEKENY SCQ--EDEEL E-SSLCELDT SAMTSSIHLL LDLSTG----
Frog_ccna1      VDEPEE-PTE TYS--IEEDC --PTLDEVDS NIVKQNIHLL LDLSA-----
Zebrafish_ccna1 IGDS------ -----VIETA TVQSV-KSTS FLLPSEL-LV LDDAVQDI--
Consistency     7874843543 46500043*45 4356739677 5577668879 *857800000

               . . . . . . . . . 210 . . . . . . . . . 220 . . . . . . . . . 230 . . . . . . . . . 240 . . . . . . . . . 250
Human_CCNA1     ---------- -------VSP MLVDSSLLSQ SEDISSLGTD VINVTEYAEE
Macaca_CCNA1    ---------- -------VSP MLVDSSLLSQ SEEISSLGTD VTNVTEYAEE
Opossum_CCNA1   ---------- -------VSP MLVDTSLQSQ FEDHLDIGTD VINVTEYAEE
Mouse_Ccna1     ---------- -------VSP MLVDPTTHAQ SEEATDFGSD VINVTEYAEE
Horse_CCNA1     ---------- -------VSP MLVDSSLHSQ SEDASDFGTD VINVTEYAEE
```

```
Platypus_CCNA1  PQGIQLKLCS RRHRSTEASP MVVDTSLQSQ LEDRLDTGPN VINVPEYAEE
Lizard_CCNA1    ---------- --------SP MSVDRSLQQQ NEELRDSNMD VMNFVEYEED
Chicken_CCNA1   ---------- --------SP MLVDTSFRSR PEDQMGDPIT LMTVGEYAED
Frog_ccna1      ---------- -------ASP MVVDTSLQAS PEDDSITDPD AVAVSEYIDE
Zebrafish_ccna1 ---------- -------GSG SFMDSSMHSL ADEEAASSED VLCVSEYAED
Consistency     0000000000 00000003*8 869*597476 4983443547 86685**798

                .........260.......270.......280.......290.......300
Human_CCNA1     IYQYLREAEI RHRPKAHYMK KQPDITEGMR TILVDWLVEV GEEYKLRAET
Macaca_CCNA1    IYQYLREAEI RHRPKAHYMK KQPDITEGMR MILVDWLVEV GEEYKLRAET
Opossum_CCNA1   IHQYLREAEM KYRPKAHYMR KQPDITEGMR TILVDWLVEV GEEYKLRAET
Mouse_Ccna1     IHRYLREAEV RHRPKAHYMR KQPDITEGMR AILVDWLVEV GEEYKLRTET
Horse_CCNA1     IHQYLREAEI RYRPKAHYMR KQPDITEGMR TILVDWLVEV GEEYKLRAET
Platypus_CCNA1  IHRYLRGVEM KHRPKAHYMQ KQPDITEAMR TILVDWLVEV GEEYKLRAET
Lizard_CCNA1    IHCYLRGAEV KYKPKPCYMR KQPDITSGMR AILVDWLVEV GEEYKLQTET
Chicken_CCNA1   IHQYLREAEV KYRPKPYYMR KQPDITTEMR AILVDWLVEV GEEYKLRTET
Frog_ccna1      IHQYLREAEL KHRPKAYYMR KQPDITSAMR TILVDWLTEV GEEYKLRTET
Zebrafish_ccna1 IHRYLRECEV KYRPKPGYMR KQPDITNCMR VILVDWLVEV GEEYKLCSET
Consistency     *86***77*7 879**65**8 ******65** 6******8** ******76**

                .........310.......320.......330.......340.......350
Human_CCNA1     LYLAVNFLDR FLSCMSVLRG KLQLVGTAAM LLASKYEEIY PPEVDEFVYI
Macaca_CCNA1    LYLAVNFLDR FLSCMSVLRG KLQLVGTAAI LLASKYEEIY PPEVDEFVYI
Opossum_CCNA1   LYLAVNFLDR FLSCMSVLRG KLQLVGTAAI LLASKYEEIY PPEVDEFVYI
Mouse_Ccna1     LYLAVNFLDR FLSCMSVLRG KLQLVGTAAI LLASKYEEIY PPDVDEFVYI
Horse_CCNA1     LYLAVNFLDR FLSCMSVLRG KLQLVGTAAI LLASKYEEIY PPEVDEFVYI
Platypus_CCNA1  LYLAINYLDR FLSCMSVLRG KLQLVGTAAI LLASKYEEIY PPEVDEFVYI
Lizard_CCNA1    LYLAVNYLDR FLSCMSVLRG KLQLVGTAAM LVAAKYEEVY PPEVDEFVYI
Chicken_CCNA1   LYLAVNYLDR FLSCMSVLRG KLQLVGTAAI LLAAKYEEIY PPEVDEFVYI
Frog_ccna1      LYLAVNYLDR FLSCMSVLRG KLQLVGTAAI LLASKYEEIY PPDVDEFVYI
Zebrafish_ccna1 LYLAVNYLDR FLSCMSVLRG KLQLVGTAAI LLAAKYEEVY PPEVDEFVYI
Consistency     ****9*8*** ********** *********8 *9*8****9* **8*******

                .........360.......370.......380.......390.......400
Human_CCNA1     TDDTYTKRQL LKMEHLLLKV LAFDLTVPTT NQFLLQYLRR QGVCVRTENL
Macaca_CCNA1    TDDTYTKRQL LKMEHLLLKV LAFDLTVPTT NQFLLQYLRR QGVCVRTENL
Opossum_CCNA1   TDDTYTKRQL LRMEHLLLKV LAFDLTVPTT NQFLLQYLQK QGVCLRTENL
Mouse_Ccna1     TDDTYTKRQL LRMEHLLLKV LAFDLTVPTT NQFLLQYLRR QGVCIRTENL
Horse_CCNA1     TDDTYTKRQL LRMEHLLLKV LAFDLTVPTT NQFLLQYLRR QGVCVRTENL
Platypus_CCNA1  TDDTYTKRQL LRMEHLLLKV LAFDLAVPTT NQFLLQYLQR QGVCLRTENL
Lizard_CCNA1    TDDTYSKKQL LRMEHLLLKV LAFDLTVPTI NQFLLQYLQR HGVSLKTENF
Chicken_CCNA1   TDDTYTKRQL LRMEHLLLKV LAFDLTVPTI NQFLLQYIHR HGVCFRTENL
Frog_ccna1      TDDTYSKKQL LRMEHLLLKV LAFDLTVPTI SQFLLQYLQR RAVSVKTEHL
Zebrafish_ccna1 TDDTYTKKQL LRMEQHLLRV LAFDMTAPTI HQFLMQYSLE EHVCARTLNL
Consistency     *****8*8** *8**88**9* ****989**6 8***9**858 67*768*888

                .........410.......420.......430.......440.......450
Human_CCNA1     AKYVAELSLL EADPFLKYLP SLIAAAAFCL ANYTVNKHFW PETLAAFTGY
Macaca_CCNA1    AKYVAELSLL EADPFLKYLP SLIAAAAFCL ANYTVNKHFW PETLAAFTGY
Opossum_CCNA1   AKYVAELSLL EADPFLKYVP SLIAAAAYCL ANYTVNRHFW PETLAAFTGY
Mouse_Ccna1     AKYVAELSLL EADPFLKYLP SLVAAAAYCL ANYIVNRHFW PETLAAFTGY
Horse_CCNA1     AKYVAELSLL EADPFLKYLP SLIAAAAYCL ANYTVNRHFW PETLAAFTGY
Platypus_CCNA1  AKYVAELSLL ETDPFLKYVP SLIAAAAYCL ANYIVNQNFW PETLAAFTGY
Lizard_CCNA1    AKYVAELSLL EVDPFLKYLP SQMAAAAYCL ANYTVNRHFW PEALAVFTGY
Chicken_CCNA1   ARYLAELSLL EADPFLKYLP SQTAAAAYCL ANYTVNRSFW PETLAAFTGY
Frog_ccna1      AMYLAELSLL DVEPFLKYVP SITAAAAYCL ANYALNKVFW PETLETFTGY
Zebrafish_ccna1 ALYLSELSLL EVDPFVQYLP SKTAAAAYCL ANYTLNGALW PENLYAFTGY
Consistency     *6*89***** 969**99*8* *56****8** ***68*658* **7*78****

                .........460.......470.......480.......490.......500
Human_CCNA1     SLSEIVPCLS ELHKAYLDIP HRPQQAIREK YKASKYLCVS LMEPPAVLLL
Macaca_CCNA1    SLSEIVPCLS ELHKAYLDIP HRPQQAIREK YKASKYLRVS LMEPPSVLLL
Opossum_CCNA1   SLSEIVPCLS ELHRACLDIP HRPQQAIREK YKASKYLHVS LMEPPAVLPL
Mouse_Ccna1     SLNEIVPCLS ELHKACLSIP HRPQQAIREK YKASKYLHVS LMEPPVVLPL
Horse_CCNA1     SLSEIVPCLS ELHKACLDIT HRPQQAIREK YKASKYMHVS LLEPPAVLPL
Platypus_CCNA1  SLREIGPCLN ELHRACLDVP HRLQQAIREK YKAPKYMHVS LMEPPVFLPL
```

```
Lizard_CCNA1       SLSEIVPCLS DLHRACLEAP HWQQQAIREK YKLAKYLEVS LLEPPLILPL
Chicken_CCNA1      SLSEIVPCLT DLHKTCLDAP HCQLQAIKEK YKQSKYLQVS LLEPPAVLPL
Frog_ccna1         TLSEITPCLS DLHQASLRAP FQAQQAIREK YKTPKYMQVS LLEMPAVLPL
Zebrafish_ccna1    SLAVIGPCLK ELHKLHLGAG SRPQQAIQEK YKSSKYHGVS QLEPVETLPL
Consistency        9*78*6***7 8**774*567 7558***8** **66**74** 88*88557*6*
```

```
                   .
Human_CCNA1        Q
Macaca_CCNA1       Q
Opossum_CCNA1      K
Mouse_Ccna1        Q
Horse_CCNA1        Q
Platypus_CCNA1     Q
Lizard_CCNA1       Q
Chicken_CCNA1      H
Frog_ccna1         N
Zebrafish_ccna1    P
Consistency        5
```

# CCND2

Unconserved 0 1 2 3 4 5 6 7 8 9 10 Conserved

```
              .........10 .........20 .........30 .........40 .........50
Human_CCND2     MELLCHEVDP VRRAVRDRNL LRDDRVLQNL LTIEERYLPQ CSYFKCVQKD
Macaca_CCND2    MELLCCEVDP VRRAVRDRNL LQDDRVLQNL LTIEERYLPQ CSYFKCVQKD
Horse_CCND2     MELLCCEVDP VRRAVPDANL LYDDRVLQNL LTIEERYLPQ CSYFKCVQKD
Mouse_Ccnd2     MELLCCEVDP VRRAVPDRNL L-EDRVLQNL LTIEERYLPQ CSYFKCVQKD
Opossum_CCND2   MELLCCEVDP IRRAVPDPSL LFDDRVLLNL LTIEERYLPQ CSYFKCVQKD
Chicken_CCND2   MELLCCEVDP MRRALPDPNL LYDDRVLHNL LTIEERYLPQ CSYFKCVQKD
Frog_ccnd2      MELLCCEGDT VRRAQADPTL LLDDRVLHNL LTVEERYLPQ CSYFKCVQKD
Platypus_CCND2  MELLCCEAEP VRRALPDPNL LNDDRVLLNL LTIEERYLPQ CSYFKCVQRD
Zebrafish_ccnd2 MELLCLEMDT IIRARPDPNL LYDDRVLQSL LTIEERFLPQ CSYFKCVQKD
Consistency     *****6*697 88**55*57* *29****59* **9***9*** ********9*

              .........60 .........70 .........80 .........90 ........100
Human_CCND2     IQPYMRRMVA TWMLEVCEEQ KCEEEVFPLA MNYLDRFLAG VPTPKSHLQL
Macaca_CCND2    IQPYMRRMVA TWMLEVCEEQ KCEEEVFPLA MNYLDRFLAG VPTPKSHLQL
Horse_CCND2     IQPYMRRMVA TWMLEVCEEQ KCEEEVFPLA MNYLDRFLAG VPTPKTHLQL
Mouse_Ccnd2     IQPYMRRMVA TWMLEVCEEQ KCEEEVFPLA MNYLDRFLAG VPTPKTHLQL
Opossum_CCND2   IQPYMRRMVA TWMLEVCEEQ KCEEEVFPLA MNYLDRFLAG VPTPKCRLQL
Chicken_CCND2   IQPFMRRMVA TWMLEVCEEQ KCEEEVFPLA MNYLDRFLAV VPTRKCHLQL
Frog_ccnd2      IQPFMRRMVA TWMLEVCEEQ RCEEEVFPLA MNYLDRFLAV IPTRKCHLQL
Platypus_CCND2  IQPHMRRMVA TWMLEVCEEQ KCEEEVFPLA MNYLDRFLAG VPTPKHHLQL
Zebrafish_ccnd2 IQPFMRRMVA TWMLEVCEEQ KCEEEVFPLA MNYLDRFLAV VPTRKCNLQL
Consistency     ***7****** ********** 9********* *********5 9**6*47***

              ........110 ........120 ........130 ........140 ........150
Human_CCND2     LGAVCMFLAS KLKETSPLTA EKLCIYTDNS IKPQELLEWE LVVLGKLKWN
Macaca_CCND2    LGAVCMFLAS KLKETSPLTA EKLCIYTDNS IKPQELLEWE LVVLGKLKWN
Horse_CCND2     LGAVCMFLAS KLKETIPLTA EKLCIYTDNS IKPQELLEWE LVVLGKLKWN
Mouse_Ccnd2     LGAVCMFLAS KLKETIPLTA EKLCIYTDNS VKPQELLEWE LVVLGKLKWN
Opossum_CCND2   LGAVCMFLAS KLKETIPLTA EKLCIYTDNS VKPQELLEWE LVVLGKLKWN
Chicken_CCND2   LGAVCMFLAS KLKETIPLTA EKLCIYTDNS IKPQELLEWE LVVLGKLKWN
Frog_ccnd2      LGAVCMFLAS KLKETIPLTA EKLCIYTDNS IKPQELLEWE LVVLGKLKWN
Platypus_CCND2  LGAVCMFLAS KLKETAPLTA EKLCIYTDNS IKPRELLEWE LVVLGKLKWN
Zebrafish_ccnd2 LGAVCMFLAS KLKETRPLTA EKLCIYTDNS IRPQELLEWE LVVLGKLKWN
Consistency     ********** *****5**** ********** 99*9****** **********

              ........160 ........170 ........180 ........190 ........200
Human_CCND2     LAAVTPHDFI EHILRKLPQQ REKLSLIRKH AQTFIALCAT DFKFAMYPPS
Macaca_CCND2    LAAVTPHDFI EHILRKLPQQ REKLSVVRKH AQTFIALCAT DFKFAMYPPS
Horse_CCND2     LAAVTPHDFI EHILRKLPQP NDKLPLIRKH AQTFIALCAT DFKFAMYPPS
Mouse_Ccnd2     LAAVTPHDFI EHILRKLPQQ KEKLSLIRKH AQTFIALCAT DFKFAMYPPS
Opossum_CCND2   LAAVTPHDFI EHILRKLPLP KDKLLLIRKH AQTFIALCAT DFNFAMYPPS
Chicken_CCND2   LAAVTPHDFI EHILRKLPLP KDKLVLIRKH AQTFIALCAT DFNFAMYPPS
Frog_ccnd2      LAAVTPHDFI EHILRKLPLP KEKLLLIRKH AQTFIALCAT DFNFAMYPPS
Platypus_CCND2  LAAVTPHDFI EHILRKLCQP RDKLLLIRKH AQTFIALCAT DFTFAMYPPS
Zebrafish_ccnd2 LAAVTPNDFI EHIMRKLPLP EDKLELIRKH VQTFIALCAT DFNFAMYPPS
Consistency     ******8*** ***9***756 67**499*** 8********* **6*******

              ........210 ........220 ........230 ........240 ........250
Human_CCND2     MIATGSVGAA ICGLQQDEEV SSLTCDALTE LLAKITNTDV DCLKACQEQI
Macaca_CCND2    MIATGSVGAA ICGLQQDEEV SSLTCDALTE LLAKITNTDV DCLKACQEQI
Horse_CCND2     MIATGSVGAA ICGLQQDEDV SSLTGDALVD LLAKITNTDV DCLKACQEQI
Mouse_Ccnd2     MIATGSVGAA ICGLQQDDEV NTLTCDALTE LLAKITHTDV DCLKACQEQI
Opossum_CCND2   MIATGSVGAA ICGLQLDSED NSLS-EHLTE LLAKITNTDV DCLKACQEQI
Chicken_CCND2   MIATGSVGAA ICGLQLDDGD RSLSGDSLTD FLAKITSTDV DCLKACQEQI
Frog_ccnd2      MIATGSVGAA ICGLQLDDGE TSFSGDSLTE HLAKITSTDV DCLKACQEQI
Platypus_CCND2  MIATGSVGAA ICGLQLDVEE SALSGDGLTD LLAKITNTDV DCLKACQEQI
Zebrafish_ccnd2 MIATGSVAAA ICGLQLNSTN HSLWGDNLTE LLAKITNTEV DVLKACQEQI
Consistency     *******8** *****58554 5886395*88 6*****7*9* *8********
```

```
               .........260.........270.........280.........290........
Human_CCND2     EAVLLNSL--  ----QQYRQD  --QRD-GSKS  EDELDQASTP  TDVRDIDL
Macaca_CCND2    EAVLLNSL--  ----QQYRQD  --QRD-GSKS  EDELDQASTP  TDVRDIDL
Horse_CCND2     EVVLLNSL--  ----QQFRQD  --QGD-GSKS  EDELDQASTP  TDVRDIDL
Mouse_Ccnd2     EALLLNSL--  ----QQFRQE  --QHNAGSKS  VEDPDQATTP  TDVRDVDL
Opossum_CCND2   EAVLVNNL--  ----RQVRQE  E-QQRNPTKT  VDELDQASTP  TDVRDINL
Chicken_CCND2   ESVLVSNL--  ----RQVRQQ  Q-QQSNPSKT  IEELDQASTP  TDVRDINL
Frog_ccnd2      ESVLVSSL--  ----RQTRQQ  A-TGSN-SKS  VNELDQASTP  TDVQDINL
Platypus_CCND2  EAVLVHNL--  ----RRVQQR  EPPPSQPSKM  VDEVDQAGTP  TDVRDINL
Zebrafish_ccnd2 ERVLMNNLRE  GRRQQQKQQQ  QEQGGQRSKA  LDDQDQSSTP  TDVRDINL
Consistency     *69*777*00  00007948*6  1073523 9*6 5785**97**  ***9*97*
```
