# Supplementary material for: Computer-Aided Identification and Design of Ligands for Multi-Targeting Inhibition of a Molecular Acute Myeloid Leukemia Network
Source: Cancers (Basel). 2024 Oct 25;16(21):3607. doi: 10.3390/cancers16213607 (PMC11544916; doi:10.3390/cancers16213607)
Supplement: Supplementary file 1 [file cancers-16-03607-s001.zip › SupplementaryMaterial_20241023/Table S4.pdf]

Table S4. List of three independent molecular dynamics simulations with DNA-bound DNMT3A with varying concentrations of procaine.

Molecular Dynamics Simulations	Number of replicates	Time (ns)
Active Site Procaine (ASP)	3	3 x 500 *ns*
Excess Procaine (EP)	3	3 x 500 *ns*
