# Supplementary figures and images for: Computer-Aided Identification and Design of Ligands for Multi-Targeting Inhibition of a Molecular Acute Myeloid Leukemia Network
Source: Cancers (Basel). 2024 Oct 25;16(21):3607. doi: 10.3390/cancers16213607 (PMC11544916; doi:10.3390/cancers16213607)

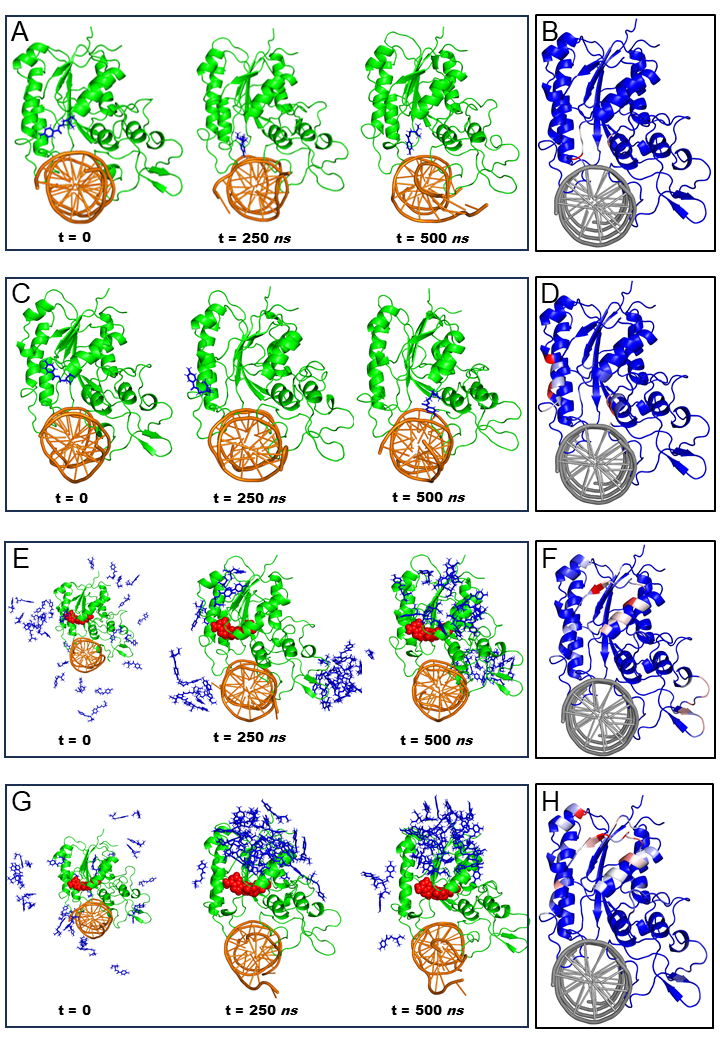

Supplement: Supplementary file 1 [file cancers-16-03607-s001.zip › SupplementaryMaterial_20241023/Figure S1.tif]

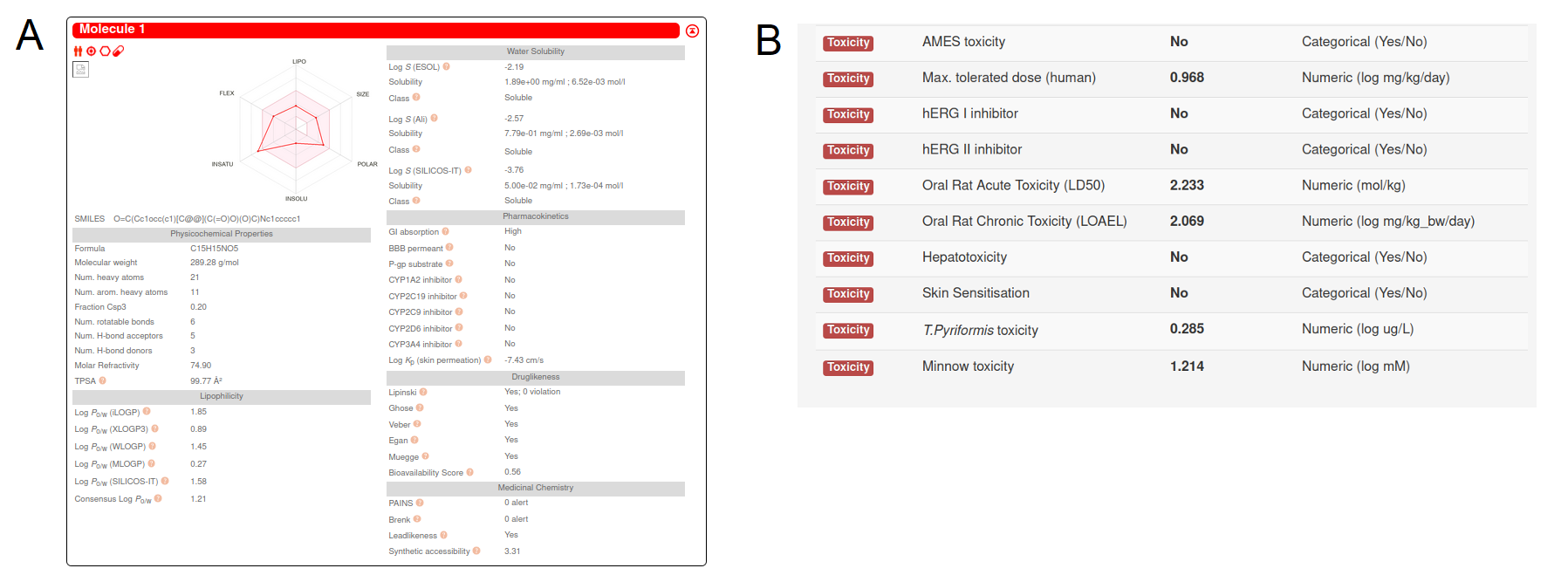

Supplement: Supplementary file 1 [file cancers-16-03607-s001.zip › SupplementaryMaterial_20241023/Figure S4.png]

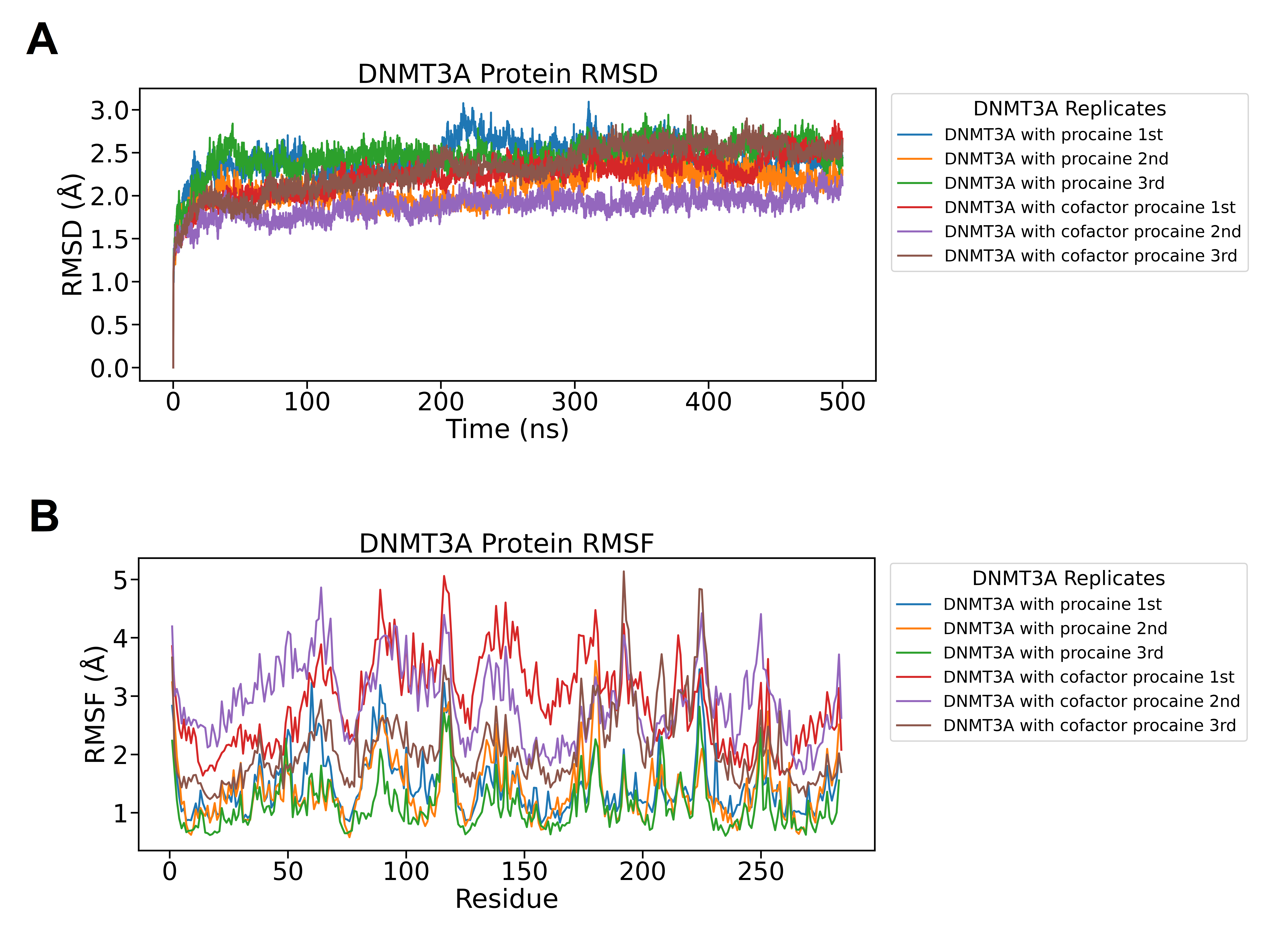

Supplement: Supplementary file 1 [file cancers-16-03607-s001.zip › SupplementaryMaterial_20241023/FigureS2.png]

## Slide 1
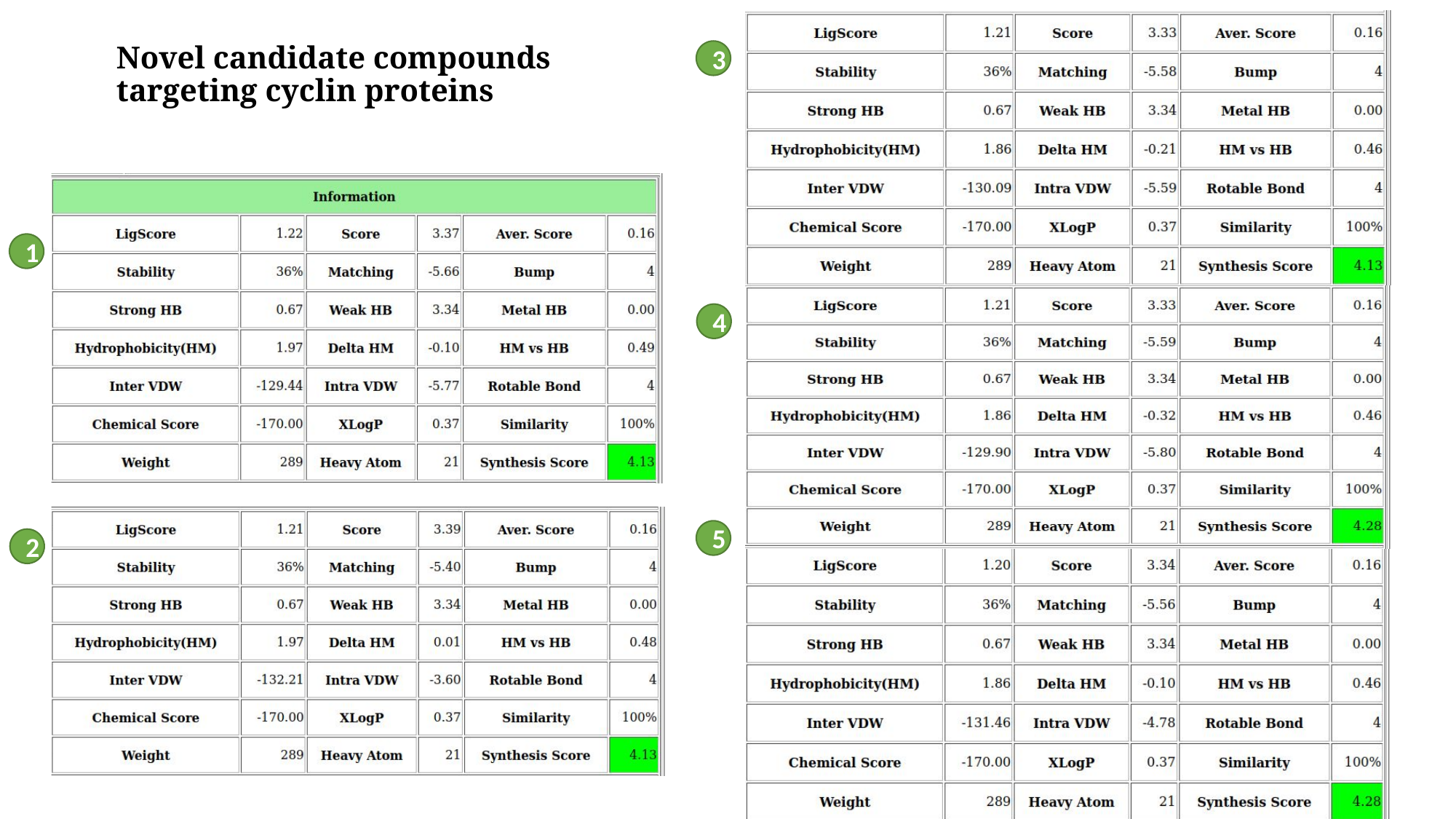

# Novel candidate compounds targeting cyclin proteins
3
1
4
5
2

Supplement: Supplementary file 1 [file cancers-16-03607-s001.zip › SupplementaryMaterial_20241023/FigureS3.pptx]
